# Supplementary material for: Multi-level inhibition of coronavirus replication by chemical ER stress
Source: Nat Commun. 2021 Sep 20;12:5536. doi: 10.1038/s41467-021-25551-1 (PMC8452654; doi:10.1038/s41467-021-25551-1)
Supplement: Supplementary file 1 — Supplementary Information [file 41467_2021_25551_MOESM1_ESM.pdf]

Supplementary Information

Supplementary Fig. 1. Opposing effects of HCoV-229E or MERS-CoV on ER stress genes at the mRNA and protein level.

Supplementary Fig. 2. PERK inhibition by GSK2656157 suppresses CoV replication.

Supplementary Fig. 3. Thapsigargin suppresses MERS-CoV and SARS-CoV-2 N protein and upregulates BiP in infected cells.

Supplementary Fig. 4. Thapsigargin suppresses Influenza A virus but not poliovirus replication.

Supplementary Fig. 5. Comparison of the antiviral effects of thapsigargin and remdesivir.

Supplementary Fig. 6. Identification of deregulated cellular pathways in MERS-CoV-infected HuH7 cells.

Supplementary Fig. 7. Identification of deregulated cellular pathways in SARS-CoV-2-infected Vero E6 cells.

Supplementary Fig. 8. Top pathways regulated by MERS-CoV, SARS-CoV-2 or thapsigargin.

Supplementary Fig. 9. Projection of thapsigargin effects on protein levels of pathway KEGG hsa04141.

Supplementary Fig. 10. Effects of bafilomycin A_1 on the viability of HCoV-229E-infected and thapsigargin-treated HuH7 cells.

Supplementary Fig. 11. Analysis of inflammatory host cell transcripts showing thapsigargin-independent uncoupling of mRNA and protein levels in HCoV-229E-infected cells.

Supplementary Table 1. List of all commercial Taqman assays and primers used in this study.

HCoV-229E / - (24 h)

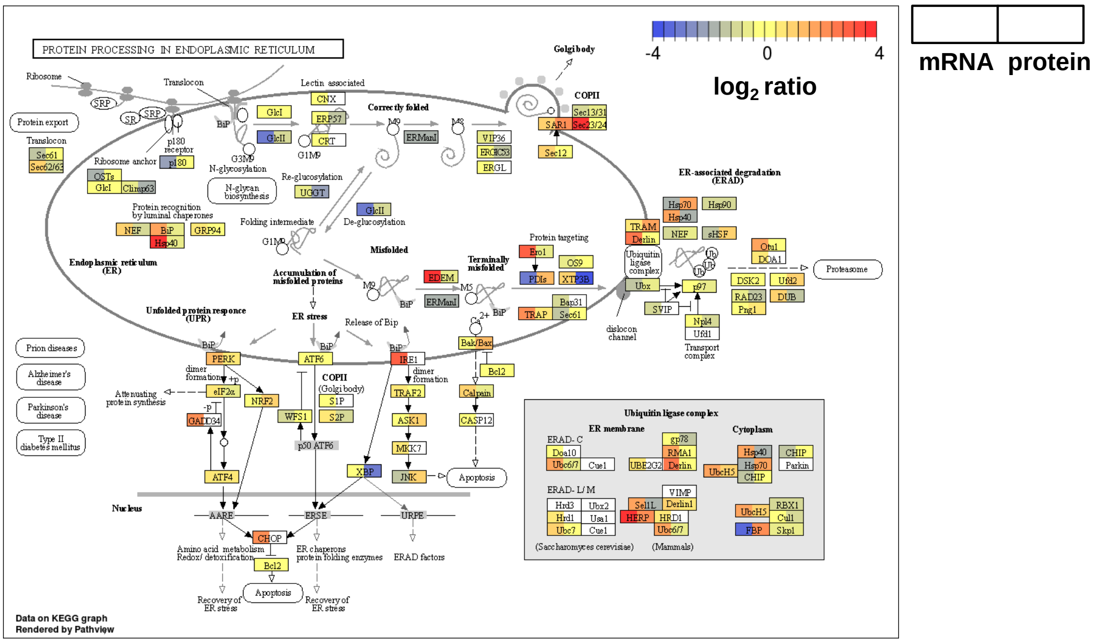

MERS-CoV / - (24 h)

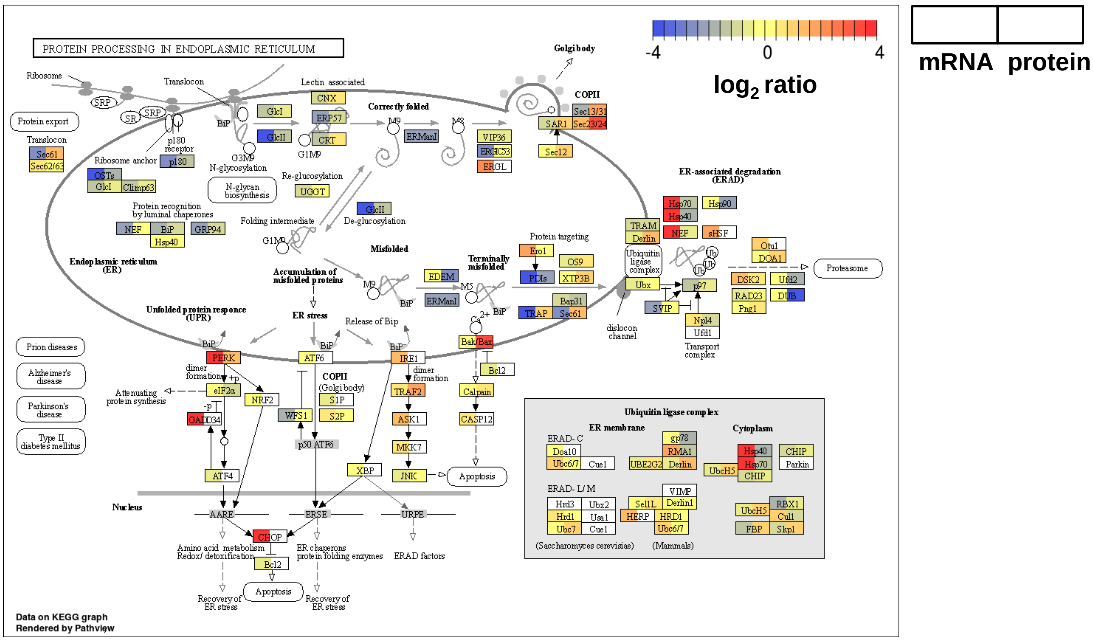

Supplementary Fig. 1. Opposing effects of HCoV-229E or MERS-CoV on ER stress genes at the mRNA and protein level.

Projection of ratio values obtained from normalized transcriptomic (by RNA-seq) and proteomic (by LC-MS/MS) data derived in parallel from HuH7 cells infected for 24 h with HCoV-229E or MERS-CoV with a MOI=1 on the components of the KEGG pathway hsa04141 “protein processing in endoplasmic reticulum”. The left side of the boxes show mRNA values, right sides show protein values.

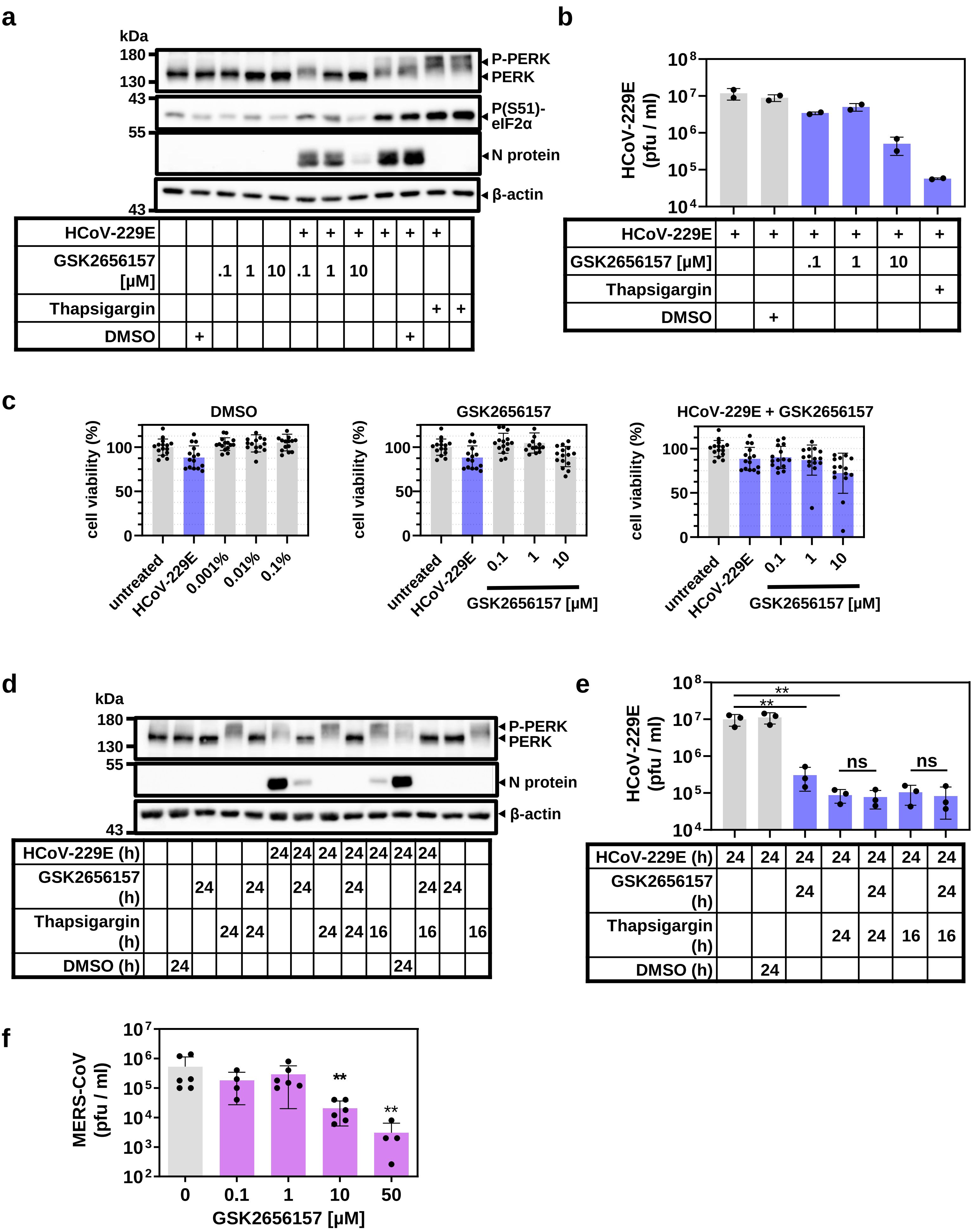
a
kDa
180
130
43
55
43
P-PERK
PERK
P(S51)-eIF2α
N protein
β-actin
HCoV-229E
GSK2656157 [µM]
.1 1 10 .1 1 10
Thapsigargin
DMSO
b
HCoV-229E (pfu / ml)
10^8
10^7
10^6
10^5
10^4
HCoV-229E
GSK2656157 [µM]
.1 1 10
Thapsigargin
DMSO
c
DMSO
GSK2656157
HCoV-229E + GSK2656157
cell viability (%)
100
50
0
untreated
HCoV-229E
0.001%
0.01%
0.1%
0.1 1 10
GSK2656157 [µM]
d
kDa
180
130
55
43
P-PERK
PERK
N protein
β-actin
HCoV-229E (h)
GSK2656157 (h)
Thapsigargin (h)
DMSO (h)
24
16
e
HCoV-229E (pfu / ml)
**
**
ns
ns
HCoV-229E (h)
GSK2656157 (h)
Thapsigargin (h)
DMSO (h)
24
16
f
MERS-CoV (pfu / ml)
10^7
10^6
10^5
10^4
10^3
10^2
**
**
0 0.1 1 10 50
GSK2656157 [µM]

Supplementary Fig. 2. PERK inhibition by GSK2656157 suppresses CoV replication.
(a) HuH7 cells were pretreated with increasing concentrations of the PERK inhibitor GSK2656157 for 30 min or with solvent (DMSO), or were left untreated. Then, cells were infected with HCoV-229E (MOI = 1) as indicated. Cell extracts were analyzed by immunoblotting for the phosphorylation or expression of the indicated proteins (one representative out of two independent experiments).
(b) Viral titers in supernatants obtained from cells treated or infected as in (a) (two biologically independent experiments).
(c) Cell viabilities (by MTS assays) of cells treated as in (a). Data points show replicate determinations of five independent experiments.
(d, e) HuH7 cells were infected and treated with the indicated combinations of GSK2656157 (10 µM) and thapsigargin (1 µM). Activation or suppression of PERK and N protein levels were determined by immunoblotting of cell extracts. (d) shows one out of two representative immunoblot experiment, (e) shows viral titers of supernatants (three biologically independent experiments).
(f) Viral titers of supernatants from HuH7 cells infected with MERS-CoV in the presence or absence of increasing concentrations of GSK2656157 (four or more biologically independent experiments).
All bar graphs show means ± s.d.; asterisks indicate p values (* $p \leq 0.05$, ** $p \leq 0.01$, *** $p \leq 0.001$, **** $p \leq 0.0001$) obtained by two-tailed unpaired t-tests (e) or Mann Withney tests (f).

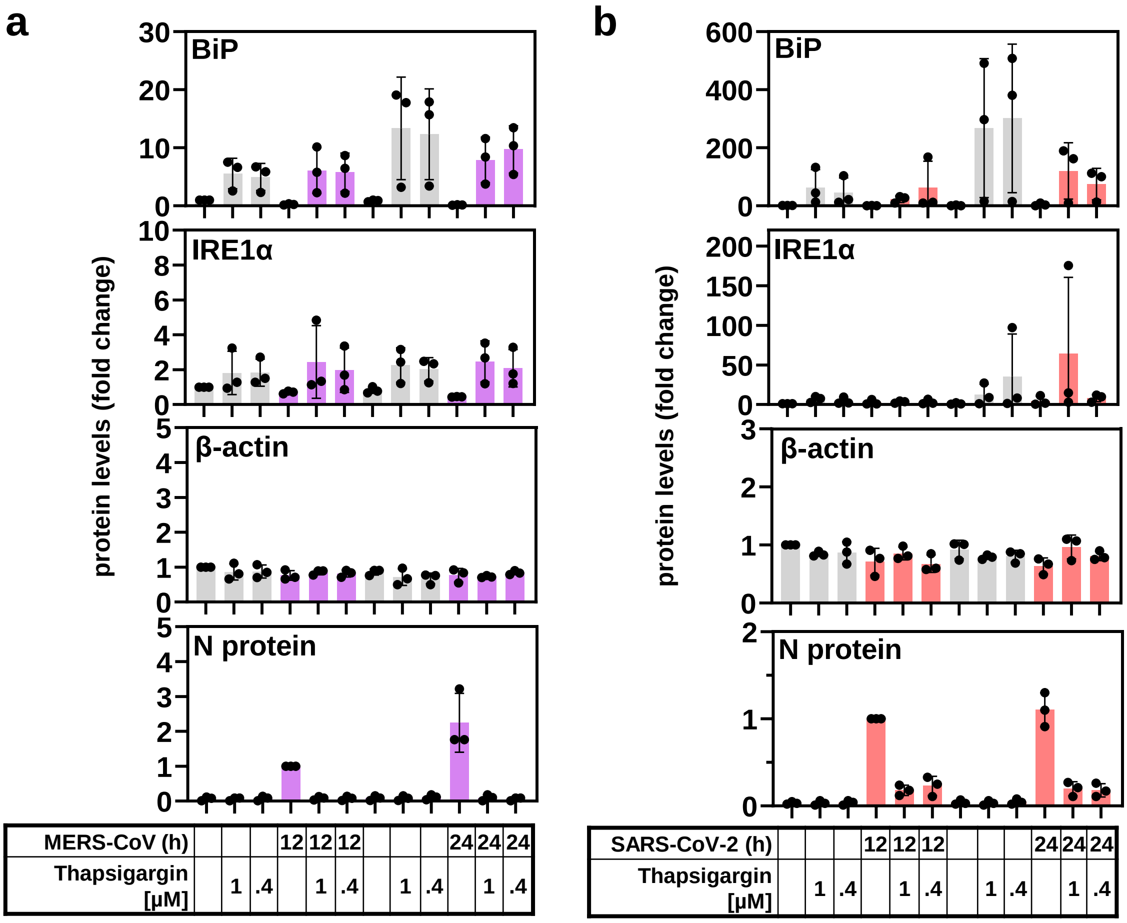

a
b
BiP
IRE1α
β-actin
N protein
protein levels (fold change)
MERS-CoV (h)
12 12 12 24 24 24
Thapsigargin [μM]
1 .4 1 .4 1 .4 1 .4
SARS-CoV-2 (h)
12 12 12 24 24 24
Thapsigargin [μM]
1 .4 1 .4 1 .4 1 .4

Supplementary Fig. 3. Thapsigargin suppresses MERS-CoV and SARS-CoV-2 N protein and upregulates BiP in infected cells.

(a, b) show the quantification of replicate immunoblot experiments performed as shown in Fig. 4g and 4h. All bar graphs show means ± s.d. (three biologically independent experiments).

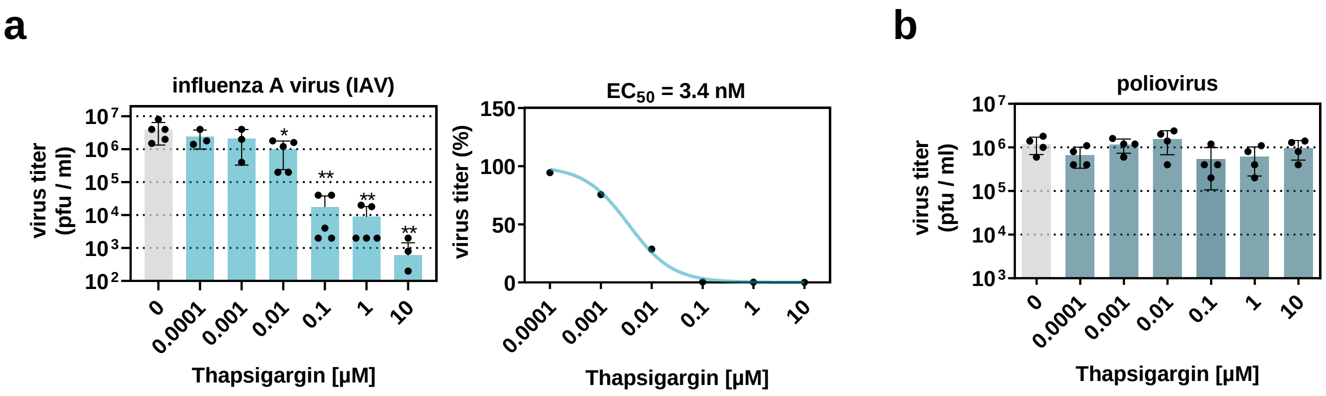
a
influenza A virus (IAV)
virus titer
(pfu / ml)
10^7
10^6
10^5
10^4
10^3
10^2
*
**
**
**
0
0.0001
0.001
0.01
0.1
1
10
Thapsigargin [µM]
EC50 = 3.4 nM
virus titer (%)
150
100
50
0
0.0001
0.001
0.01
0.1
1
10
Thapsigargin [µM]
b
poliovirus
virus titer
(pfu / ml)
10^7
10^6
10^5
10^4
10^3
0
0.0001
0.001
0.01
0.1
1
10
Thapsigargin [µM]

Supplementary Fig. 4. Thapsigargin suppresses Influenza A virus but not poliovirus replication.

(a) Shown are viral titers and the EC_{50} (for IAV) of A549 cells infected with IAV virus (strain A/Thailand/1(KAN-1)/2004, H5N1) at an MOI of 0.01 for 24 h (three or more biologically independent experiments).

(b) Viral titers of Vero E6 cells infected with poliovirus at an MOI of 0.1 for 24 h and treated with thapsigargin as indicated (four biologically independent experiments).

All bar graphs show means ± s.d.; asterisks indicate p values (* $p \leq 0.05$, ** $p \leq 0.01$, *** $p \leq 0.001$, **** $p \leq 0.0001$) obtained by two-tailed unpaired t-tests.

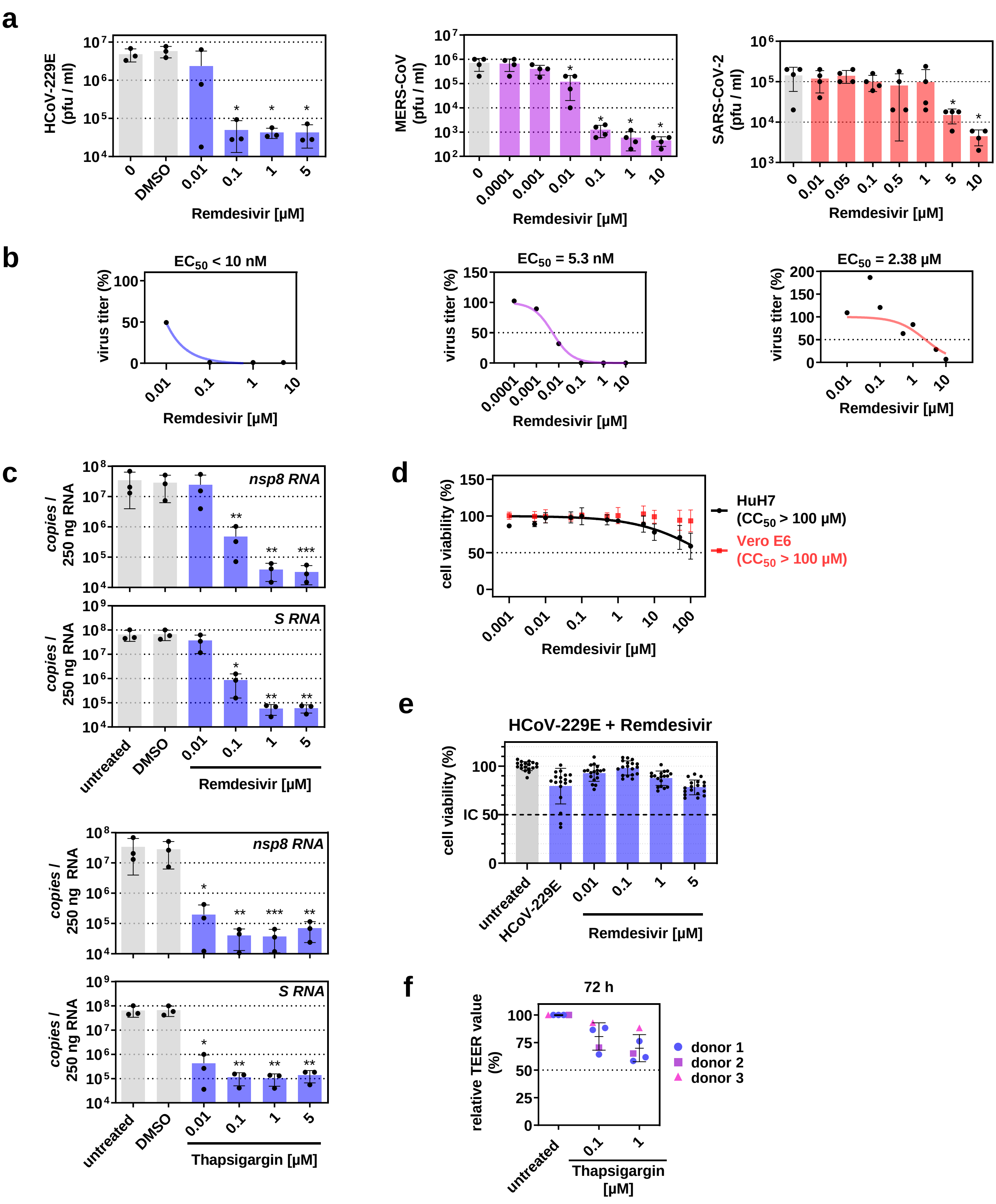
a
HCoV-229E (pfu / ml)
0
DMSO
0.01
0.1
1
5
Remdesivir [µM]
MERS-CoV (pfu / ml)
0
0.0001
0.001
0.01
0.1
1
10
Remdesivir [µM]
SARS-CoV-2 (pfu / ml)
0
0.01
0.05
0.1
0.5
1
5
10
Remdesivir [µM]
b
EC50 < 10 nM
virus titer (%)
Remdesivir [µM]
EC50 = 5.3 nM
virus titer (%)
Remdesivir [µM]
EC50 = 2.38 µM
virus titer (%)
Remdesivir [µM]
c
copies / 250 ng RNA
nsp8 RNA
S RNA
untreated
DMSO
0.01
0.1
1
5
Remdesivir [µM]
nsp8 RNA
S RNA
Thapsigargin [µM]
d
cell viability (%)
HuH7 (CC50 > 100 µM)
Vero E6 (CC50 > 100 µM)
0.001
0.01
0.1
1
10
100
Remdesivir [µM]
e
HCoV-229E + Remdesivir
cell viability (%)
IC 50
untreated
HCoV-229E
0.01
0.1
1
5
Remdesivir [µM]
f
72 h
relative TEER value (%)
donor 1
donor 2
donor 3
untreated
0.1
1
Thapsigargin [µM]

Supplementary Fig. 5. Comparison of the antiviral effects of thapsigargin and remdesivir.
(a, b) HuH7 cells infected with HCoV-229E (MOI of 1) or MERS-CoV (MOI of 0.5) or Vero E6 cells infected with SARS-CoV-2 (MOI of 0.5) for 24 h with an were treated with increasing concentrations of remdesivir and viral titers in the supernatants obtained from these cells (a) were used to calculate the EC_{50} values shown in (b). Data represent three (HCoV-229E) or four (MERS-CoV, SARS-CoV-2) biologically independent experiments.
(c) Direct comparison of the effects of remdesivir or thapsigargin on the synthesis of viral RNAs in cells infected with HCoV-229E as described in (a) (three biologically independent experiments).
(d) Cytotoxicity of remdesivir in untreated HuH7 or Vero E6 cells. Data show CC_{50} estimations obtained from two or more biologically independent experiments.
(e) Cytotoxicity of remdesivir in HuH7 cells infected with HCoV-229E (24 h, MOI of 1) as determined by MTS assays. Data points show replicate determinations representing six biologically independent experiments.
(f) Viability of differentiated NHBE cells treated for 72 h with two doses of thapsigargin was compared to untreated cells by TEER assay (three biologically independent experiments).
All bar graphs show means ± s.d.; asterisks indicate p values (* $p \leq 0.05$, ** $p \leq 0.01$, *** $p \leq 0.001$, **** $p \leq 0.0001$) obtained by two-tailed unpaired t-tests.

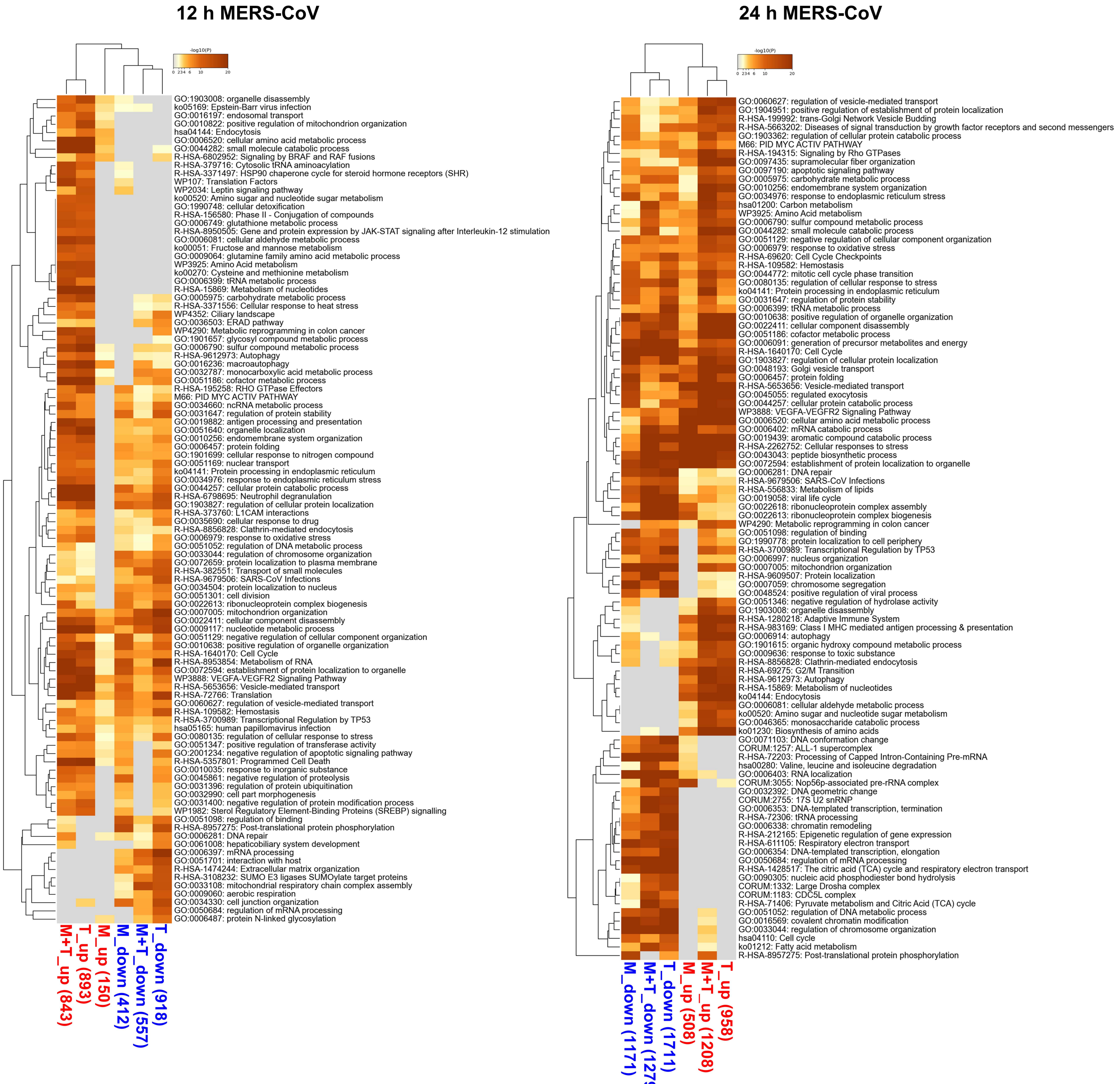

12 h MERS-CoV
-log10(P)
0 2 3 4 6 10 20
GO:1903008: organelle disassembly
ko05169: Epstein-Barr virus infection
GO:0016197: endosomal transport
GO:0010822: positive regulation of mitochondrion organization
hsa04144: Endocytosis
GO:0006520: cellular amino acid metabolic process
GO:0044282: small molecule catabolic process
R-HSA-6802952: Signaling by BRAF and RAF fusions
R-HSA-379716: Cytosolic tRNA aminoacylation
R-HSA-3371497: HSP90 chaperone cycle for steroid hormone receptors (SHR)
WP107: Translation Factors
WP2034: Leptin signaling pathway
ko00520: Amino sugar and nucleotide sugar metabolism
GO:1990748: cellular detoxification
R-HSA-156580: Phase II - Conjugation of compounds
GO:0006749: glutathione metabolic process
R-HSA-8950505: Gene and protein expression by JAK-STAT signaling after Interleukin-12 stimulation
GO:0006081: cellular aldehyde metabolic process
ko00051: Fructose and mannose metabolism
GO:0009064: glutamine family amino acid metabolic process
WP3925: Amino Acid metabolism
ko00270: Cysteine and methionine metabolism
GO:0006399: tRNA metabolic process
R-HSA-15869: Metabolism of nucleotides
GO:0005975: carbohydrate metabolic process
R-HSA-3371556: Cellular response to heat stress
WP4352: Ciliary landscape
GO:0036503: ERAD pathway
WP4290: Metabolic reprogramming in colon cancer
GO:1901657: glycosyl compound metabolic process
GO:0006790: sulfur compound metabolic process
R-HSA-9612973: Autophagy
GO:0016236: macroautophagy
GO:0032787: monocarboxylic acid metabolic process
GO:0051186: cofactor metabolic process
R-HSA-195258: RHO GTPase Effectors
M66: PID MYC ACTIV PATHWAY
GO:0034660: ncRNA metabolic process
GO:0031647: regulation of protein stability
GO:0019882: antigen processing and presentation
GO:0051640: organelle localization
GO:0010256: endomembrane system organization
GO:0006457: protein folding
GO:1901699: cellular response to nitrogen compound
GO:0051169: nuclear transport
ko04141: Protein processing in endoplasmic reticulum
GO:0034976: response to endoplasmic reticulum stress
GO:0044257: cellular protein catabolic process
R-HSA-6798695: Neutrophil degranulation
GO:1903827: regulation of cellular protein localization
R-HSA-373760: L1CAM interactions
GO:0035690: cellular response to drug
R-HSA-8856828: Clathrin-mediated endocytosis
GO:0006979: response to oxidative stress
GO:0051052: regulation of DNA metabolic process
GO:0033044: regulation of chromosome organization
GO:0072659: protein localization to plasma membrane
R-HSA-382551: Transport of small molecules
R-HSA-9679506: SARS-CoV Infections
GO:0034504: protein localization to nucleus
GO:0051301: cell division
GO:0022613: ribonucleoprotein complex biogenesis
GO:0007005: mitochondrion organization
GO:0022411: cellular component disassembly
GO:0009117: nucleotide metabolic process
GO:0051129: negative regulation of cellular component organization
GO:0010638: positive regulation of organelle organization
R-HSA-1640170: Cell Cycle
R-HSA-8953854: Metabolism of RNA
GO:0072594: establishment of protein localization to organelle
WP3888: VEGFA-VEGFR2 Signaling Pathway
R-HSA-5653656: Vesicle-mediated transport
R-HSA-72766: Translation
GO:0060627: regulation of vesicle-mediated transport
R-HSA-109582: Hemostasis
R-HSA-3700989: Transcriptional Regulation by TP53
hsa05165: human papillomavirus infection
GO:0080135: regulation of cellular response to stress
GO:0051347: positive regulation of transferase activity
GO:2001234: negative regulation of apoptotic signaling pathway
R-HSA-5357801: Programmed Cell Death
GO:0010035: response to inorganic substance
GO:0045861: negative regulation of proteolysis
GO:0031396: regulation of protein ubiquitination
GO:0032990: cell part morphogenesis
GO:0031400: negative regulation of protein modification process
WP1982: Sterol Regulatory Element-Binding Proteins (SREBP) signalling
GO:0051098: regulation of binding
R-HSA-8957275: Post-translational protein phosphorylation
GO:0006281: DNA repair
GO:0061008: hepaticobiliary system development
GO:0006397: mRNA processing
GO:0051701: interaction with host
R-HSA-1474244: Extracellular matrix organization
R-HSA-3108232: SUMO E3 ligases SUMOylate target proteins
GO:0033108: mitochondrial respiratory chain complex assembly
GO:0009060: aerobic respiration
GO:0034330: cell junction organization
GO:0050684: regulation of mRNA processing
GO:0006487: protein N-linked glycosylation
M+T_up (843)
T_up (893)
M_up (150)
M_down (412)
M+T_down (557)
T_down (918)
24 h MERS-CoV
-log10(P)
0 2 3 4 6 10 20
GO:0060627: regulation of vesicle-mediated transport
GO:1904951: positive regulation of establishment of protein localization
R-HSA-199992: trans-Golgi Network Vesicle Budding
R-HSA-5663202: Diseases of signal transduction by growth factor receptors and second messengers
GO:1903362: regulation of cellular protein catabolic process
M66: PID MYC ACTIV PATHWAY
R-HSA-194315: Signaling by Rho GTPases
GO:0097435: supramolecular fiber organization
GO:0097190: apoptotic signaling pathway
GO:0005975: carbohydrate metabolic process
GO:0010256: endomembrane system organization
GO:0034976: response to endoplasmic reticulum stress
hsa01200: Carbon metabolism
WP3925: Amino Acid metabolism
GO:0006790: sulfur compound metabolic process
GO:0044282: small molecule catabolic process
GO:0051129: negative regulation of cellular component organization
GO:0006979: response to oxidative stress
R-HSA-69620: Cell Cycle Checkpoints
R-HSA-109582: Hemostasis
GO:0044772: mitotic cell cycle phase transition
GO:0080135: regulation of cellular response to stress
ko04141: Protein processing in endoplasmic reticulum
GO:0031647: regulation of protein stability
GO:0006399: tRNA metabolic process
GO:0010638: positive regulation of organelle organization
GO:0022411: cellular component disassembly
GO:0051186: cofactor metabolic process
GO:0006091: generation of precursor metabolites and energy
R-HSA-1640170: Cell Cycle
GO:1903827: regulation of cellular protein localization
GO:0048193: Golgi vesicle transport
GO:0006457: protein folding
R-HSA-5653656: Vesicle-mediated transport
GO:0045055: regulated exocytosis
GO:0044257: cellular protein catabolic process
WP3888: VEGFA-VEGFR2 Signaling Pathway
GO:0006520: cellular amino acid metabolic process
GO:0006402: mRNA catabolic process
GO:0019439: aromatic compound catabolic process
R-HSA-2262752: Cellular responses to stress
GO:0043043: peptide biosynthetic process
GO:0072594: establishment of protein localization to organelle
GO:0006281: DNA repair
R-HSA-9679506: SARS-CoV Infections
R-HSA-556833: Metabolism of lipids
GO:0019058: viral life cycle
GO:0022618: ribonucleoprotein complex assembly
GO:0022613: ribonucleoprotein complex biogenesis
WP4290: Metabolic reprogramming in colon cancer
GO:0051098: regulation of binding
GO:1990778: protein localization to cell periphery
R-HSA-3700989: Transcriptional Regulation by TP53
GO:0006997: nucleus organization
GO:0007005: mitochondrion organization
R-HSA-9609507: Protein localization
GO:0007059: chromosome segregation
GO:0048524: positive regulation of viral process
GO:0051346: negative regulation of hydrolase activity
GO:1903008: organelle disassembly
R-HSA-1280218: Adaptive Immune System
R-HSA-983169: Class I MHC mediated antigen processing & presentation
GO:0006914: autophagy
GO:1901615: organic hydroxy compound metabolic process
GO:0009636: response to toxic substance
R-HSA-8856828: Clathrin-mediated endocytosis
R-HSA-69275: G2/M Transition
R-HSA-9612973: Autophagy
R-HSA-15869: Metabolism of nucleotides
ko04144: Endocytosis
GO:0006081: cellular aldehyde metabolic process
ko00520: Amino sugar and nucleotide sugar metabolism
GO:0046365: monosaccharide catabolic process
ko01230: Biosynthesis of amino acids
GO:0071103: DNA conformation change
CORUM:1257: ALL-1 supercomplex
R-HSA-72203: Processing of Capped Intron-Containing Pre-mRNA
hsa00280: Valine, leucine and isoleucine degradation
GO:0006403: RNA localization
CORUM:3055: Nop56p-associated pre-rRNA complex
GO:0032392: DNA geometric change
CORUM:2755: 17S U2 snRNP
GO:0006353: DNA-templated transcription, termination
R-HSA-72306: tRNA processing
GO:0006338: chromatin remodeling
R-HSA-212165: Epigenetic regulation of gene expression
R-HSA-611105: Respiratory electron transport
GO:0006354: DNA-templated transcription, elongation
GO:0050684: regulation of mRNA processing
R-HSA-1428517: The citric acid (TCA) cycle and respiratory electron transport
GO:0090305: nucleic acid phosphodiester bond hydrolysis
CORUM:1332: Large Drosha complex
CORUM:1183: CDC5L complex
R-HSA-71406: Pyruvate metabolism and Citric Acid (TCA) cycle
GO:0051052: regulation of DNA metabolic process
GO:0016569: covalent chromatin modification
GO:0033044: regulation of chromosome organization
hsa04110: Cell cycle
ko01212: Fatty acid metabolism
R-HSA-8957275: Post-translational protein phosphorylation
M_down (1171)
M+T_down (1279)
T_down (1711)
M_up (508)
M+T_up (1208)
T_up (958)

Supplementary Fig. 6. Identification of deregulated cellular pathways in MERS-CoV-infected HuH7 cells.

Top 100 overrepresented pathways containing up- or downregulated DEPs (ratio > 0, p value of $-\log_{10}(p) \geq 1.3$) for the 12 h p.i. and 24 h p.i. time points of MERS-CoV-infected cells based on gene IDs derived from protein IDs. Blue and red colors indicate differentially expressed proteins as shown in Fig. 6a, b, e. See the legends of Fig. 6 and Methods for details.

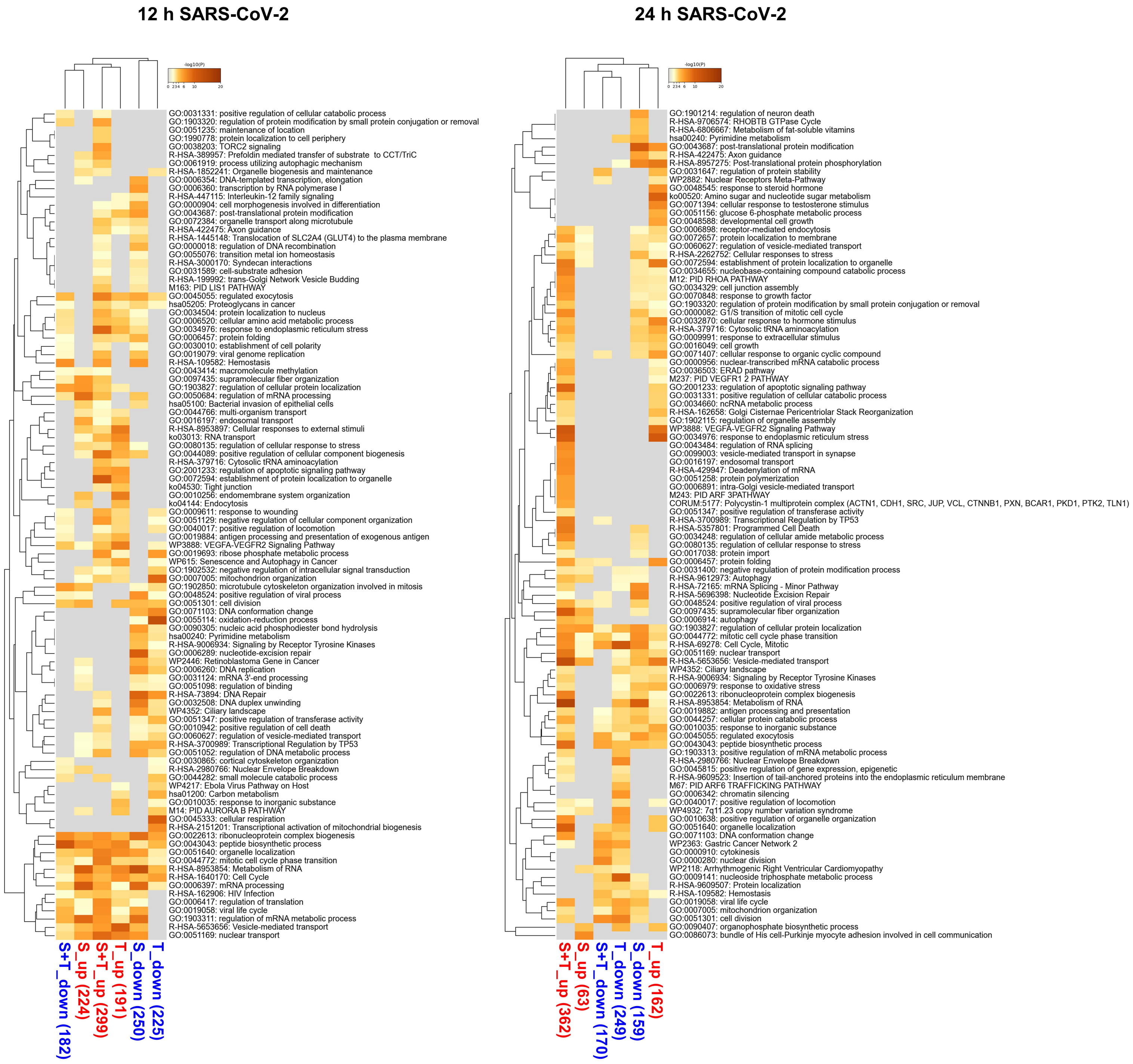
12 h SARS-CoV-2
-log10(P)
0 2 3 4 6 10 20
GO:0031331: positive regulation of cellular catabolic process
GO:1903320: regulation of protein modification by small protein conjugation or removal
GO:0051235: maintenance of location
GO:1990778: protein localization to cell periphery
GO:0038203: TORC2 signaling
R-HSA-389957: Prefoldin mediated transfer of substrate to CCT/TriC
GO:0061919: process utilizing autophagic mechanism
R-HSA-1852241: Organelle biogenesis and maintenance
GO:0006354: DNA-templated transcription, elongation
GO:0006360: transcription by RNA polymerase I
R-HSA-447115: Interleukin-12 family signaling
GO:0000904: cell morphogenesis involved in differentiation
GO:0043687: post-translational protein modification
GO:0072384: organelle transport along microtubule
R-HSA-422475: Axon guidance
R-HSA-1445148: Translocation of SLC2A4 (GLUT4) to the plasma membrane
GO:0000018: regulation of DNA recombination
GO:0055076: transition metal ion homeostasis
R-HSA-3000170: Syndecan interactions
GO:0031589: cell-substrate adhesion
R-HSA-199992: trans-Golgi Network Vesicle Budding
M163: PID LIS1 PATHWAY
GO:0045055: regulated exocytosis
hsa05205: Proteoglycans in cancer
GO:0034504: protein localization to nucleus
GO:0006520: cellular amino acid metabolic process
GO:0034976: response to endoplasmic reticulum stress
GO:0006457: protein folding
GO:0030010: establishment of cell polarity
GO:0019079: viral genome replication
R-HSA-109582: Hemostasis
GO:0043414: macromolecule methylation
GO:0097435: supramolecular fiber organization
GO:1903827: regulation of cellular protein localization
GO:0050684: regulation of mRNA processing
hsa05100: Bacterial invasion of epithelial cells
GO:0044766: multi-organism transport
GO:0016197: endosomal transport
R-HSA-8953897: Cellular responses to external stimuli
ko03013: RNA transport
GO:0080135: regulation of cellular response to stress
GO:0044089: positive regulation of cellular component biogenesis
R-HSA-379716: Cytosolic tRNA aminoacylation
GO:2001233: regulation of apoptotic signaling pathway
GO:0072594: establishment of protein localization to organelle
ko04530: Tight junction
GO:0010256: endomembrane system organization
ko04144: Endocytosis
GO:0009611: response to wounding
GO:0051129: negative regulation of cellular component organization
GO:0040017: positive regulation of locomotion
GO:0019884: antigen processing and presentation of exogenous antigen
WP3888: VEGFA-VEGFR2 Signaling Pathway
GO:0019693: ribose phosphate metabolic process
WP615: Senescence and Autophagy in Cancer
GO:1902532: negative regulation of intracellular signal transduction
GO:0007005: mitochondrion organization
GO:1902850: microtubule cytoskeleton organization involved in mitosis
GO:0048524: positive regulation of viral process
GO:0051301: cell division
GO:0071103: DNA conformation change
GO:0055114: oxidation-reduction process
GO:0090305: nucleic acid phosphodiester bond hydrolysis
hsa00240: Pyrimidine metabolism
R-HSA-9006934: Signaling by Receptor Tyrosine Kinases
GO:0006289: nucleotide-excision repair
WP2446: Retinoblastoma Gene in Cancer
GO:0006260: DNA replication
GO:0031124: mRNA 3'-end processing
GO:0051098: regulation of binding
R-HSA-73894: DNA Repair
GO:0032508: DNA duplex unwinding
WP4352: Ciliary landscape
GO:0051347: positive regulation of transferase activity
GO:0010942: positive regulation of cell death
GO:0060627: regulation of vesicle-mediated transport
R-HSA-3700989: Transcriptional Regulation by TP53
GO:0051052: regulation of DNA metabolic process
GO:0030865: cortical cytoskeleton organization
R-HSA-2980766: Nuclear Envelope Breakdown
GO:0044282: small molecule catabolic process
WP4217: Ebola Virus Pathway on Host
hsa01200: Carbon metabolism
GO:0010035: response to inorganic substance
M14: PID AURORA B PATHWAY
GO:0045333: cellular respiration
R-HSA-2151201: Transcriptional activation of mitochondrial biogenesis
GO:0022613: ribonucleoprotein complex biogenesis
GO:0043043: peptide biosynthetic process
GO:0051640: organelle localization
GO:0044772: mitotic cell cycle phase transition
R-HSA-8953854: Metabolism of RNA
R-HSA-1640170: Cell Cycle
GO:0006397: mRNA processing
R-HSA-162906: HIV Infection
GO:0006417: regulation of translation
GO:0019058: viral life cycle
GO:1903311: regulation of mRNA metabolic process
R-HSA-5653656: Vesicle-mediated transport
GO:0051169: nuclear transport
S+T_down (182)
S_up (224)
S+T_up (299)
T_up (191)
S_down (250)
T_down (225)
24 h SARS-CoV-2
-log10(P)
0 2 3 4 6 10 20
GO:1901214: regulation of neuron death
R-HSA-9706574: RHOBTB GTPase Cycle
R-HSA-6806667: Metabolism of fat-soluble vitamins
hsa00240: Pyrimidine metabolism
GO:0043687: post-translational protein modification
R-HSA-422475: Axon guidance
R-HSA-8957275: Post-translational protein phosphorylation
GO:0031647: regulation of protein stability
WP2882: Nuclear Receptors Meta-Pathway
GO:0048545: response to steroid hormone
ko00520: Amino sugar and nucleotide sugar metabolism
GO:0071394: cellular response to testosterone stimulus
GO:0051156: glucose 6-phosphate metabolic process
GO:0048588: developmental cell growth
GO:0006898: receptor-mediated endocytosis
GO:0072657: protein localization to membrane
GO:0060627: regulation of vesicle-mediated transport
R-HSA-2262752: Cellular responses to stress
GO:0072594: establishment of protein localization to organelle
GO:0034655: nucleobase-containing compound catabolic process
M12: PID RHOA PATHWAY
GO:0034329: cell junction assembly
GO:0070848: response to growth factor
GO:1903320: regulation of protein modification by small protein conjugation or removal
GO:0000082: G1/S transition of mitotic cell cycle
GO:0032870: cellular response to hormone stimulus
R-HSA-379716: Cytosolic tRNA aminoacylation
GO:0009991: response to extracellular stimulus
GO:0016049: cell growth
GO:0071407: cellular response to organic cyclic compound
GO:0000956: nuclear-transcribed mRNA catabolic process
GO:0036503: ERAD pathway
M237: PID VEGFR1 2 PATHWAY
GO:2001233: regulation of apoptotic signaling pathway
GO:0031331: positive regulation of cellular catabolic process
GO:0034660: ncRNA metabolic process
R-HSA-162658: Golgi Cisternae Pericentriolar Stack Reorganization
GO:1902115: regulation of organelle assembly
WP3888: VEGFA-VEGFR2 Signaling Pathway
GO:0034976: response to endoplasmic reticulum stress
GO:0043484: regulation of RNA splicing
GO:0099003: vesicle-mediated transport in synapse
GO:0016197: endosomal transport
R-HSA-429947: Deadenylation of mRNA
GO:0051258: protein polymerization
GO:0006891: intra-Golgi vesicle-mediated transport
M243: PID ARF 3PATHWAY
CORUM:5177: Polycystin-1 multiprotein complex (ACTN1, CDH1, SRC, JUP, VCL, CTNNB1, PXN, BCAR1, PKD1, PTK2, TLN1)
GO:0051347: positive regulation of transferase activity
R-HSA-3700989: Transcriptional Regulation by TP53
R-HSA-5357801: Programmed Cell Death
GO:0034248: regulation of cellular amide metabolic process
GO:0080135: regulation of cellular response to stress
GO:0017038: protein import
GO:0006457: protein folding
GO:0031400: negative regulation of protein modification process
R-HSA-9612973: Autophagy
R-HSA-72165: mRNA Splicing - Minor Pathway
R-HSA-5696398: Nucleotide Excision Repair
GO:0048524: positive regulation of viral process
GO:0097435: supramolecular fiber organization
GO:0006914: autophagy
GO:1903827: regulation of cellular protein localization
GO:0044772: mitotic cell cycle phase transition
R-HSA-69278: Cell Cycle, Mitotic
GO:0051169: nuclear transport
R-HSA-5653656: Vesicle-mediated transport
WP4352: Ciliary landscape
R-HSA-9006934: Signaling by Receptor Tyrosine Kinases
GO:0006979: response to oxidative stress
GO:0022613: ribonucleoprotein complex biogenesis
R-HSA-8953854: Metabolism of RNA
GO:0019882: antigen processing and presentation
GO:0044257: cellular protein catabolic process
GO:0010035: response to inorganic substance
GO:0045055: regulated exocytosis
GO:0043043: peptide biosynthetic process
GO:1903313: positive regulation of mRNA metabolic process
R-HSA-2980766: Nuclear Envelope Breakdown
GO:0045815: positive regulation of gene expression, epigenetic
R-HSA-9609523: Insertion of tail-anchored proteins into the endoplasmic reticulum membrane
M67: PID ARF6 TRAFFICKING PATHWAY
GO:0006342: chromatin silencing
GO:0040017: positive regulation of locomotion
WP4932: 7q11.23 copy number variation syndrome
GO:0010638: positive regulation of organelle organization
GO:0051640: organelle localization
GO:0071103: DNA conformation change
WP2363: Gastric Cancer Network 2
GO:0000910: cytokinesis
GO:0000280: nuclear division
WP2118: Arrhythmogenic Right Ventricular Cardiomyopathy
GO:0009141: nucleoside triphosphate metabolic process
R-HSA-9609507: Protein localization
R-HSA-109582: Hemostasis
GO:0019058: viral life cycle
GO:0007005: mitochondrion organization
GO:0051301: cell division
GO:0090407: organophosphate biosynthetic process
GO:0086073: bundle of His cell-Purkinje myocyte adhesion involved in cell communication
S+T_up (362)
S_up (63)
S+T_down (170)
T_down (249)
S_down (159)
T_up (162)

Supplementary Fig. 7. Identification of deregulated cellular pathways in SARS-CoV-2-infected Vero E6 cells.

Top 100 overrepresented pathways containing up- or downregulated DEPs (ratio > 0, p value of $-\log_{10}(p) \geq 1.3$) for the 12 h p.i. and 24 h p.i. time points of SARS-CoV-2-CoV-infected cells based on gene IDs derived from protein IDs. Blue and red colors indicate differentially expressed proteins as shown in Fig. 6c, d, e. See the legends of Fig. 6 and Methods for details.

a

condition	time	GO	description	DEPs↑	DEPs↓
MERS-CoV	12h	GO:0009117	nucleotide metabolic process	-7.9	-5.0
		GO:0016236	macroautophagy	-5.9	
		GO:0022411	cellular component disassembly	-5.7	-10.1
		WP3888	VEGFA-VEGFR2 Signaling Pathway	-5.4	-8.1
		hsa04144	Endocytosis	-4.8	
		R-HSA-8957275	Post-translational protein phosphorylation		-14.8
		R-HSA-8953854	Metabolism of RNA		-13.9
		GO:0051129	negative regulation of cellular component organization		-13.0
		GO:0072594	establishment of protein localization to organelle		-13.0
		GO:0010638	positive regulation of organelle organization		-12.8
MERS-CoV	24h	R-HSA-2262752	Cellular responses to stress	-33.4	-6.6
		GO:0043043	peptide biosynthetic process	-32.5	-13.0
		GO:0006402	mRNA catabolic process	-28.7	-3.9
		GO:0019439	aromatic compound catabolic process	-27.2	-6.2
		GO:0006520	cellular amino acid metabolic process	-22.8	-3.2
		R-HSA-72203	Processing of Capped Intron-Containing Pre-mRNA	-3.3	-32.5
		GO:1903827	regulation of cellular protein localization	-7.7	-24.2
		GO:0050684	regulation of mRNA processing		-24.1
		R-HSA-1640170	Cell Cycle	-14.8	-23.6
		GO:0007005	mitochondrion organization		-22.6
SARS-CoV-2	12h	GO:0006397	mRNA processing	-11.0	-9.2
		R-HSA-8953854	Metabolism of RNA	-10.1	-12.7
		GO:1903311	regulation of mRNA metabolic process	-9.2	-8.7
		GO:0050684	regulation of mRNA processing	-8.7	-3.9
		GO:0043043	peptide biosynthetic process	-7.0	
		R-HSA-8953854	Metabolism of RNA	-10.1	-12.7
		GO:0006289	nucleotide-excision repair		-10.0
		R-HSA-73894	DNA Repair		-9.6
		GO:0006397	mRNA processing	-11.0	-9.2
		GO:0022613	ribonucleoprotein complex biogenesis	-5.2	-9.0
SARS-CoV-2	24h	GO:0086073	..His cell-Purkinje myocyte adhesion in communication	-6.83	
		GO:1903827	regulation of cellular protein localization	-5.9	-6.3
		R-HSA-5653656	Vesicle-mediated transport	-5.2	-4.6
		GO:0097435	supramolecular fiber organization	-5.0	
		GO:0090407	organophosphate biosynthetic process	-4.19	
		GO:0043687	post-translational protein modification		-10.5
		R-HSA-8953854	Metabolism of RNA		-10.0
		R-HSA-8957275	Post-translational protein phosphorylation		-7.2
		R-HSA-5696398	Nucleotide Excision Repair		-7.1
		R-HSA-69278	Cell Cycle, Mitotic		-7.0
Thapsigargin HuH7	12h	GO:0006520	cellular amino acid metabolic process	-49.0	
		GO:0051186	cofactor metabolic process	-43.3	-7.2
		R-HSA-5653656	Vesicle-mediated transport	-42.7	-6.4
		GO:0009117	nucleotide metabolic process	-38.9	-17.5
		R-HSA-5357801	Programmed Cell Death	-28.9	-4.8
		GO:0006397	mRNA processing		-24.7
		R-HSA-8953854	Metabolism of RNA	-13.7	-23.7
		GO:0007005	mitochondrion organization	-8.5	-20.5
		R-HSA-72766	Translation	-13.5	-17.5
		GO:0009117	nucleotide metabolic process	-38.9	-17.5
Thapsigargin HuH7	24h	R-HSA-5653656	Vesicle-mediated transport	-59.1	-6.0
		GO:0006520	cellular amino acid metabolic process	-34.5	-6.8
		GO:0051186	cofactor metabolic process	-34.5	-14.7
		ko04144	Endocytosis	-31.1	
		WP3888	VEGFA-VEGFR2 Signaling Pathway	-30.9	-6.7
		R-HSA-72203	Processing of Capped Intron-Containing Pre-mRNA		-100.0
		GO:0022613	ribonucleoprotein complex biogenesis	-3.1	-71.0
		GO:0006403	RNA localization		-49.6
		GO:0050684	regulation of mRNA processing		-46.6
		R-HSA-1640170	Cell Cycle	-14.9	-40.6
Thapsigargin Vero E6	12h	R-HSA-5653656	Vesicle-mediated transport	-10.4	
		R-HSA-8953897	Cellular responses to external stimuli	-8.1	
		GO:0010256	endomembrane system organization	-8.0	
		WP3888	VEGFA-VEGFR2 Signaling Pathway	-7.8	
		GO:0051169	nuclear transport	-7.4	
		GO:0055114	oxidation-reduction process		-13.5
		GO:0045333	cellular respiration		-10.2
		GO:0007005	mitochondrion organization	-3.1	-9.4
		GO:0071103	DNA conformation change		-7.1
		R-HSA-2151201	Transcriptional activation of mitochondrial biogenesis		-6.9
Thapsigargin Vero E6	24h	GO:0034976	response to endoplasmic reticulum stress	-11.3	
		ko00520	Amino sugar and nucleotide sugar metabolism	-9.8	
		WP3888	VEGFA-VEGFR2 Signaling Pathway	-8.9	
		R-HSA-8957275	Post-translational protein phosphorylation	-8.2	
		GO:0072594	establishment of protein localization to organelle	-8.2	
		R-HSA-69278	Cell Cycle, Mitotic		-11.9
		GO:0009141	nucleoside triphosphate metabolic process		-10.2
		GO:0051301	cell division		-7.44
		R-HSA-2980766	Nuclear Envelope Breakdown		-6.9
		WP4932	7q11.23 copy number variation syndrome		-6.1

top10 pathways

DNA
RNA
metabolism
localization

enrichment log_{10} p value

0 -20

b

	GO	description	virus DEPs↑	virus DEPs↓	Thapsigargin DEPs↑	Thapsigargin DEPs↓
Thapsigargin (52)	GO:1901657	glycosyl compound metabolic process			-16.7	
	ko00270	Cysteine and methionine metabolism			-15.2	
	GO:0032787	monocarboxylic acid metabolic process			-14.7	-6.7
	GO:0046365	monosaccharide catabolic process			-13.5	
	GO:0006749	glutathione metabolic process			-12.1	
	GO:0009064	glutamine family amino acid metabolic process			-11.7	
	GO:1990748	cellular detoxification			-11.5	
	R-HSA-8950505	.JAK-STAT signaling after Interleukin-12 stimulation			-11.4	
	ko00051	Fructose and mannose metabolism			-11.0	
	R-HSA-156580	Phase II - Conjugation of compounds			-10.5	
	CORUM:1332	Large Drosha complex				-15.9
	R-HSA-71406	Pyruvate metabolism and Citric Acid (TCA) cycle				-13.6
	GO:0055114	oxidation-reduction process				-13.5
	GO:0033108	mitochondrial respiratory chain complex assembly				-12.9
	R-HSA-3108232	SUMO E3 ligases SUMOylate target proteins				-12.6
	GO:0009060	aerobic respiration				-11.5
	GO:0009141	nucleoside triphosphate metabolic process				-10.2
	GO:0045333	cellular respiration				-10.2
	CORUM:1183	CDC5L complex				-9.3
	GO:0061008	hepaticobiliary system development				-7.5
Thapsigargin, MERS-CoV, SARS-CoV-2 (36)	R-HSA-2262752	Cellular responses to stress	-33.4	-6.6	-24.6	-12.8
	GO:0043043	peptide biosynthetic process	-32.5	-13.0	-17.8	-30.4
	R-HSA-5653656	Vesicle-mediated transport	-20.2	-17.1	-59.1	-6.0
	R-HSA-1640170	Cell Cycle	-14.8	-23.6	-14.9	-40.6
	GO:0045055	regulated exocytosis	-13.5	-15.3	-20.2	-5.5
	GO:0022613	ribonucleoprotein complex biogenesis	-13.4	-15.5	-3.1	-71.0
	ko04144	Endocytosis	-13.4		-31.1	
	GO:0006397	mRNA processing	-11.0	-9.2		
	GO:0044257	cellular protein catabolic process	-10.3	-15.2	-19.0	-11.1
	GO:0006457	protein folding	-10.3	-18.7	-13.4	-17.0
	GO:1903827	regulation of cellular protein localization	-7.7	-24.2	-17.3	-20.1
	GO:0050684	regulation of mRNA processing		-24.1		-46.6
	R-HSA-1640170	Cell Cycle	-14.8	-23.6	-14.9	-40.6
	GO:0007005	mitochondrion organization		-22.6	-11.0	-27.1
	GO:0006457	protein folding	-10.3	-18.7	-13.4	-17.0
	R-HSA-5653656	Vesicle-mediated transport	-20.2	-17.1	-59.1	-6.0
	R-HSA-8957275	Post-translational protein phosphorylation		-16.3		-5.7
	GO:0022613	ribonucleoprotein complex biogenesis	-13.4	-15.5	-3.1	-71.0
	GO:0045055	regulated exocytosis	-13.5	-15.3	-20.2	-5.5
	GO:0044257	cellular protein catabolic process	-10.3	-15.2	-19.0	-11.1

enrichment log_{10} p value

0 -20

Supplementary Fig. 8. Top pathways regulated by MERS-CoV, SARS-CoV-2 or thapsigargin.
(a) Top ten enriched pathways containing up- or downregulated DEPs extracted from the 100 enriched deregulated pathways shown in Fig. S6 / Fig. S7. Colors indicate highly common categories.
(b) Top 20 pathways enriched with thapsigargin alone or jointly by MERS-CoV, SARS-CoV-2 and thapsigargin according to the Venn diagram shown in Fig. 6f. See the legends of Fig. 6 and Methods for details.

Pathway KEGG: hsa04141

MERS-CoV / - (24 h)

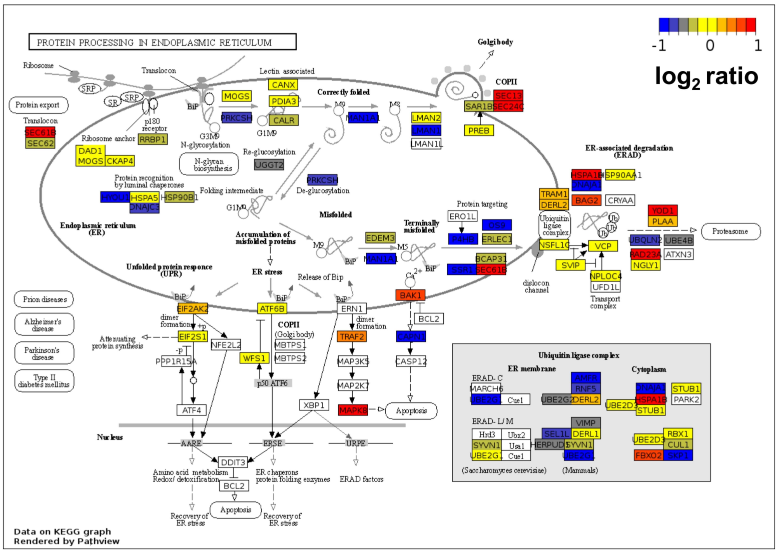

MERS-CoV + Thapsigargin / MERS-CoV (24 h)

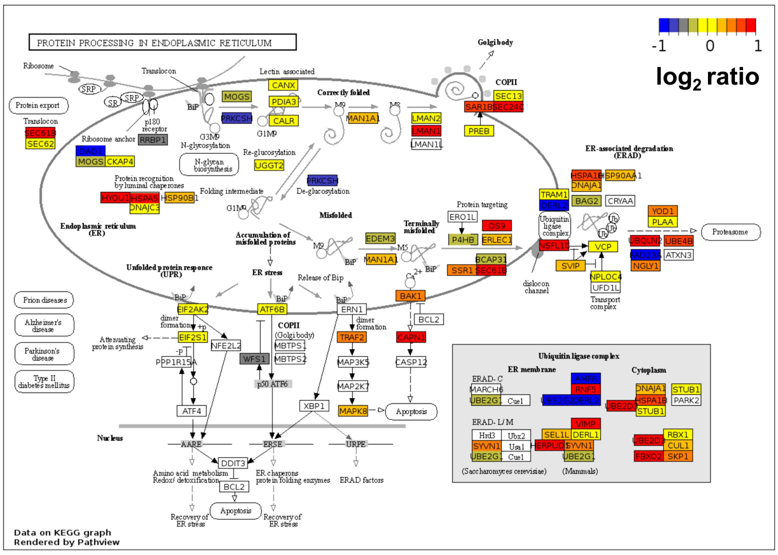

SARS-CoV-2 / - (24 h)

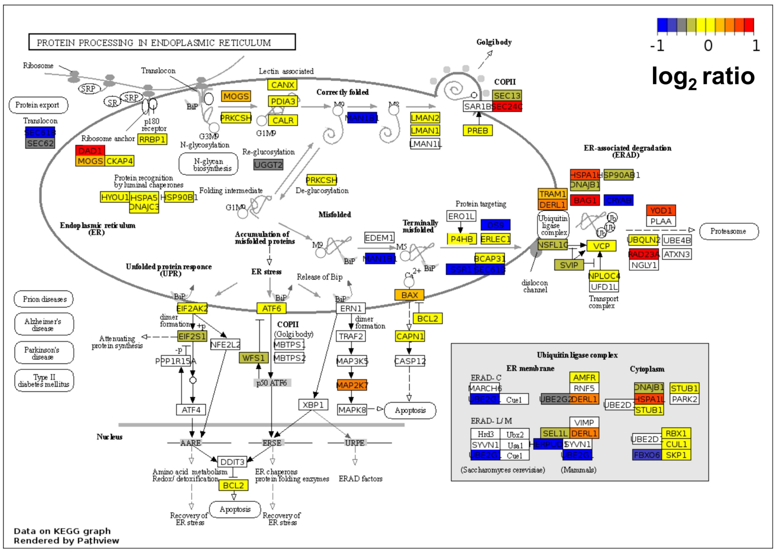

SARS-CoV-2 + Thapsigargin / SARS-CoV-2 (24 h)

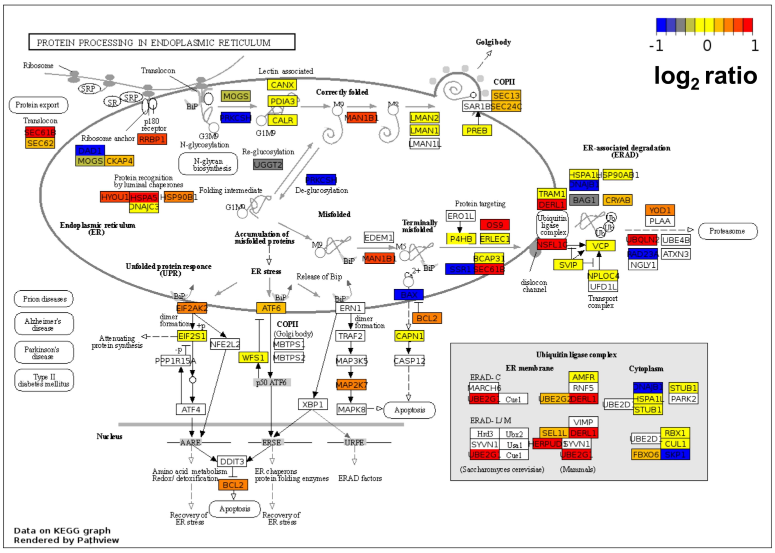

Supplementary Fig. 9. Projection of thapsigargin effects on protein levels of pathway KEGG hsa04141.

Mean ratio values of all pathway components measured by LC-MS/MS in untreated cells and 24 h p.i. were projected on the KEGG hsa04141 pathway map (left graphs). The right graphs show the corresponding changes imposed by thapsigargin treatment of infected cells.

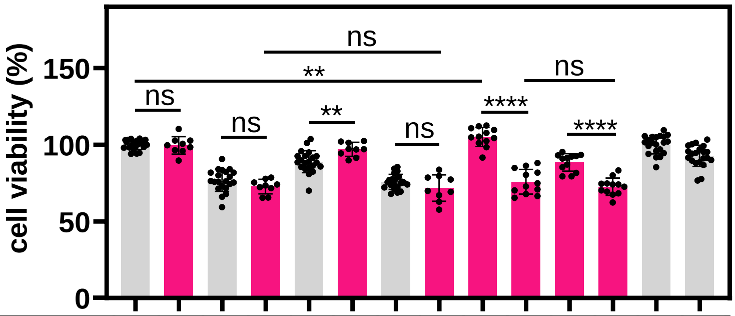

	1	2	3	4	5	6	7	8	9	10	11	12	13	14
HCoV-229E (24 h)					+	+	+	+			+	+		+
Bafilomycin A_1 (h)		28		28		28		28	16	16	16	16		
Thapsigargin (24 h)			+	+			+	+		+		+		
DMSO (28 h)													+	+

Supplementary Fig. 10. Effects of bafilomycin A_1 on the viability of HCoV-229E-infected and thapsigargin-treated HuH7 cells.

Experiments were performed as described in the legend of Fig. 8. Cell viability was determined by MTS assay. Data points show replicate determinations representing three (28 h bafilomycin A_1 treatment conditions) or four (16 h bafilomycin A_1 treatment conditions) biologically independent experiments. The bar graph shows means ± s.d.; asterisks indicate p values (* $p \leq 0.05$, ** $p \leq 0.01$, *** $p \leq 0.001$, **** $p \leq 0.0001$) obtained by two-tailed unpaired t-tests.

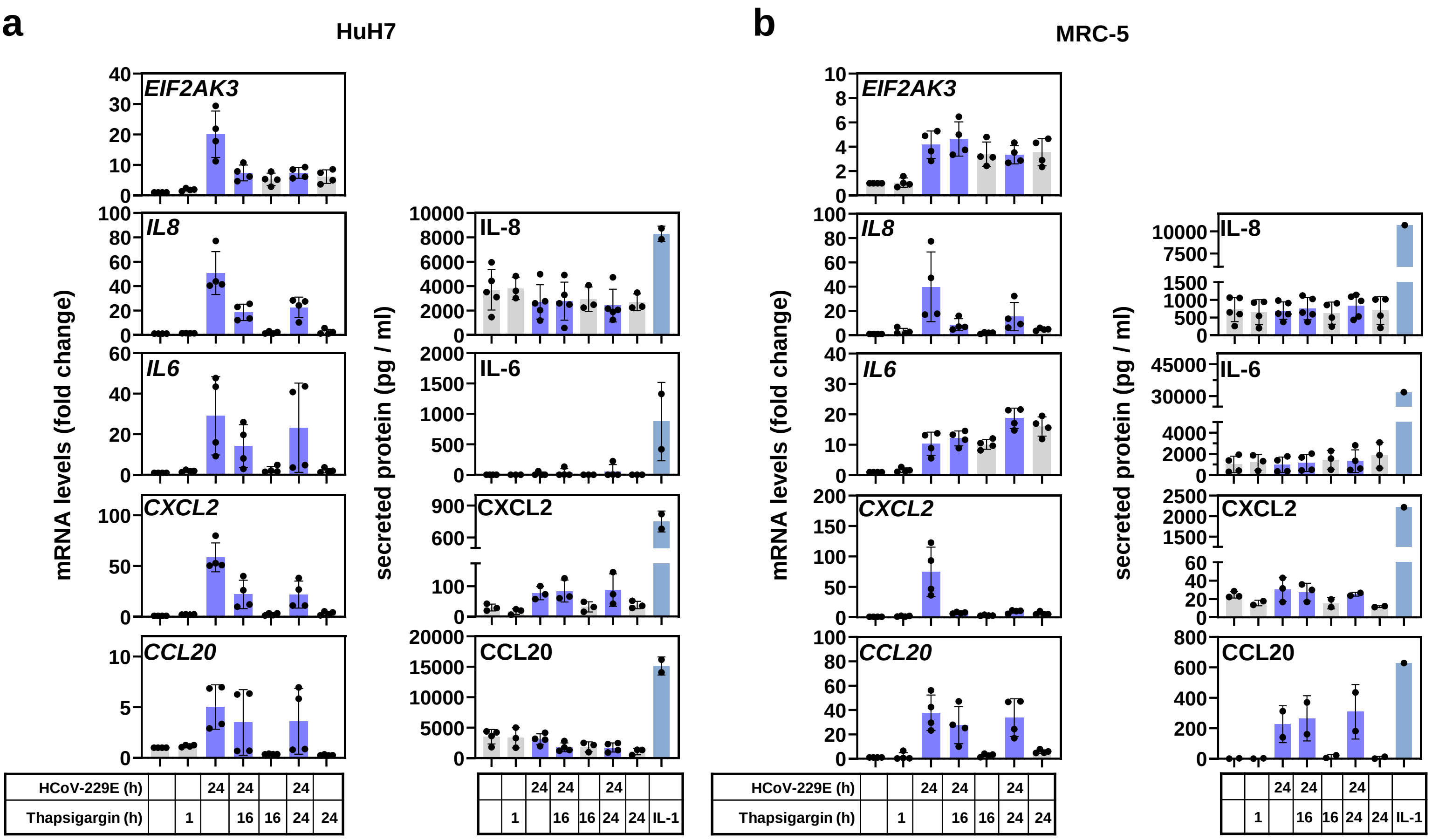
a
HuH7
b
MRC-5
mRNA levels (fold change)
secreted protein (pg / ml)
EIF2AK3
IL8
IL6
CXCL2
CCL20
IL-8
IL-6
CXCL2
CCL20
HCoV-229E (h)
Thapsigargin (h)
IL-1

Supplementary Fig. 11. Analysis of inflammatory host cell transcripts showing thapsigargin-independent uncoupling of mRNA and protein levels in HCoV-229E-infected cells.

HuH7 (a) or MRC-5 (b) cells were infected as described in Fig. 2b. Total RNA was used to analyze expression of inflammatory transcripts by RT-qPCR (left graphs) and the secretion of the corresponding proteins by ELISA (right graphs). Additionally, expression of the *EIF2AK3* mRNA encoding the PERK protein kinase was determined. PERK protein levels of HuH7 cells are shown in Fig. 2e-f. All bar graphs show means ± s.d.. Data points show individual values from two or more biologically independent experiments with the exception of IL-1 treatments which were used as positive controls for one or two experiments as indicated.

Table 1: Commercial Taqman assays used for RT-qPCR

transcript	length of PCR product (bp)	assay ID	source
GUSB	81	Hs99999908_m1	Applied Biosystems / Thermo Fisher Scientific
IL6	95	Hs00174131_m1	Applied Biosystems / Thermo Fisher Scientific
IL8 (CXCL8)	101	Hs00174103_m1	Applied Biosystems / Thermo Fisher Scientific
CXCL2	68	Hs00236966_m1	Applied Biosystems / Thermo Fisher Scientific
CCL20	81	Hs00171125_m1	Applied Biosystems / Thermo Fisher Scientific

Table 2: Additional primers designed for RT-qPCR

transcript	forward sequence	reverse sequence
EIF2AK3	5'-AGAGATTGAGACTGCGTGGC-3'	5'-TCCCAAATACCTCTGGTTTGCT-3'
HCoV-229E S RNA	5‘-TTTCAGGTGATGCTCACATACC-3‘	5‘-ACAAACTCACGAACTGTCTTAGG-3‘
HCoV-229E nsp8 RNA	5‘-GCTGTTGCAAATGGTTCCTCAC-3‘	5‘-GATGCACATTCTTACCATCATTATCC-3‘

Supplementary Table 1. List of all commercial Taqman assays and primers used in this study.
