# Supplementary material for: Multi-level inhibition of coronavirus replication by chemical ER stress
Source: Nat Commun. 2021 Sep 20;12:5536. doi: 10.1038/s41467-021-25551-1 (PMC8452654; doi:10.1038/s41467-021-25551-1)

## Slide 1
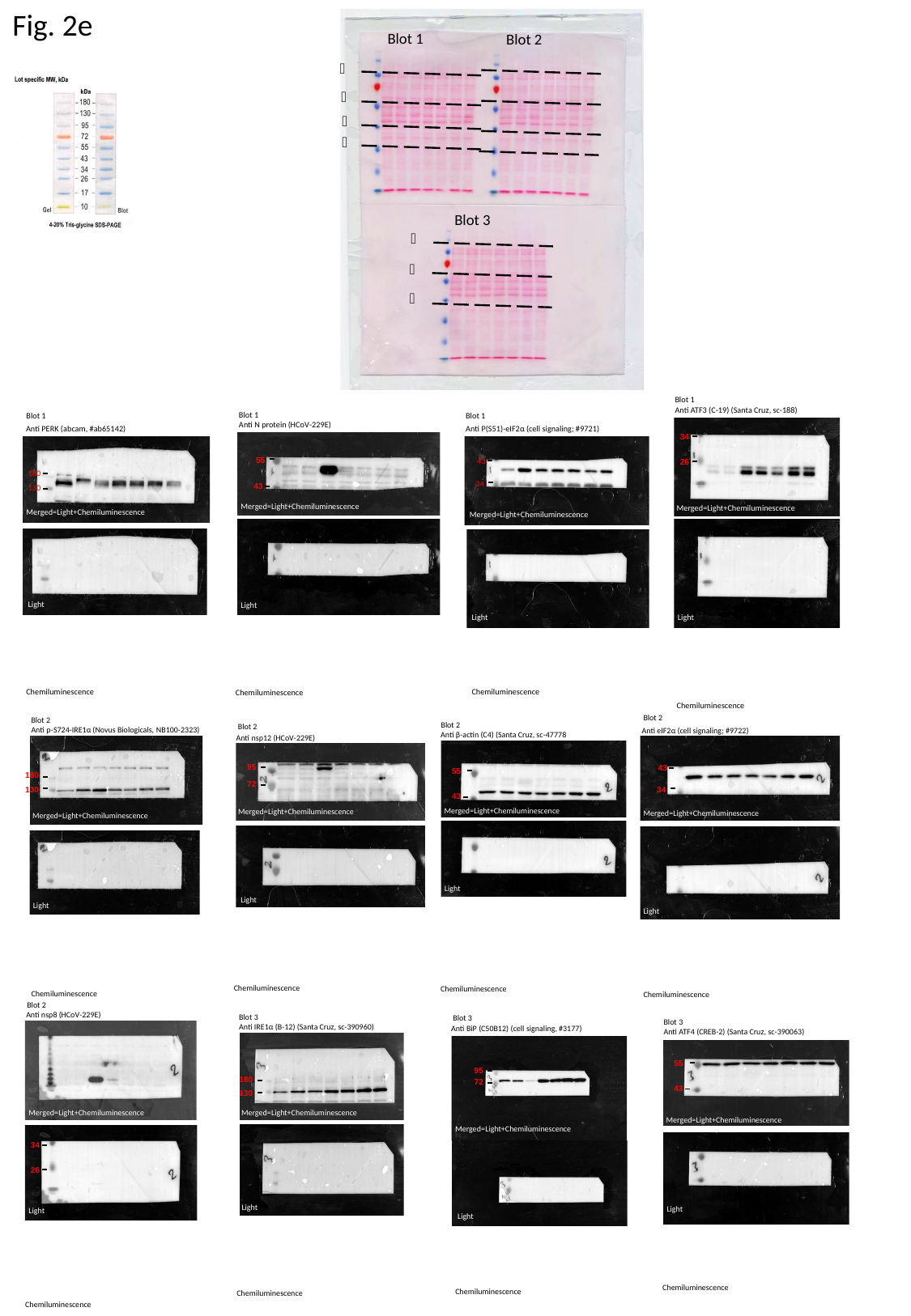

Fig. 2e

## Slide 2
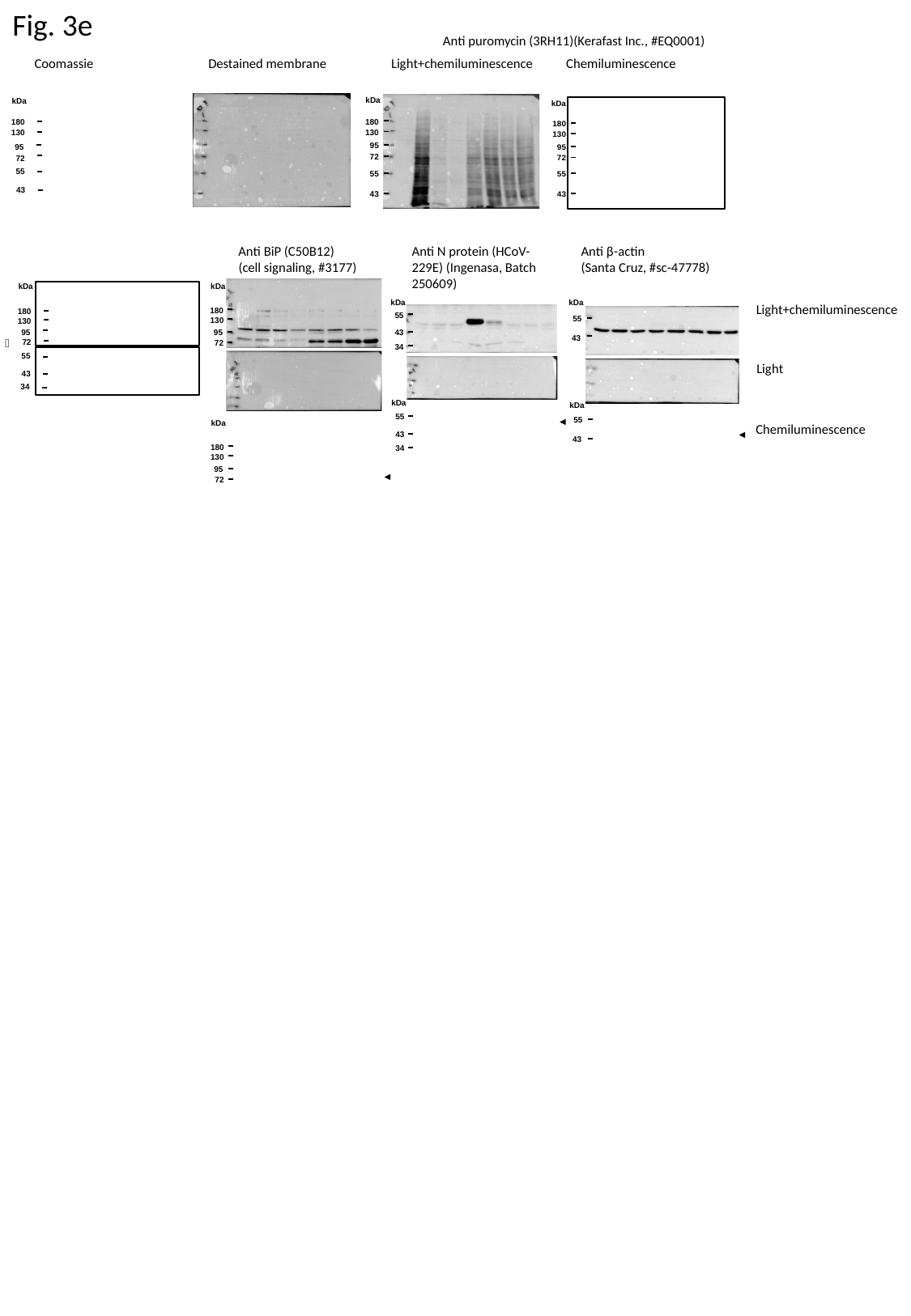

Fig. 3e
Anti puromycin (3RH11)(Kerafast Inc., #EQ0001)
Coomassie
Destained membrane
Light+chemiluminescence
Chemiluminescence
Anti BiP (C50B12)
(cell signaling, #3177)
Anti N protein (HCoV-229E) (Ingenasa, Batch 250609)
Anti β-actin
(Santa Cruz, #sc-47778)
Light+chemiluminescence
Light
Chemiluminescence

## Slide 3
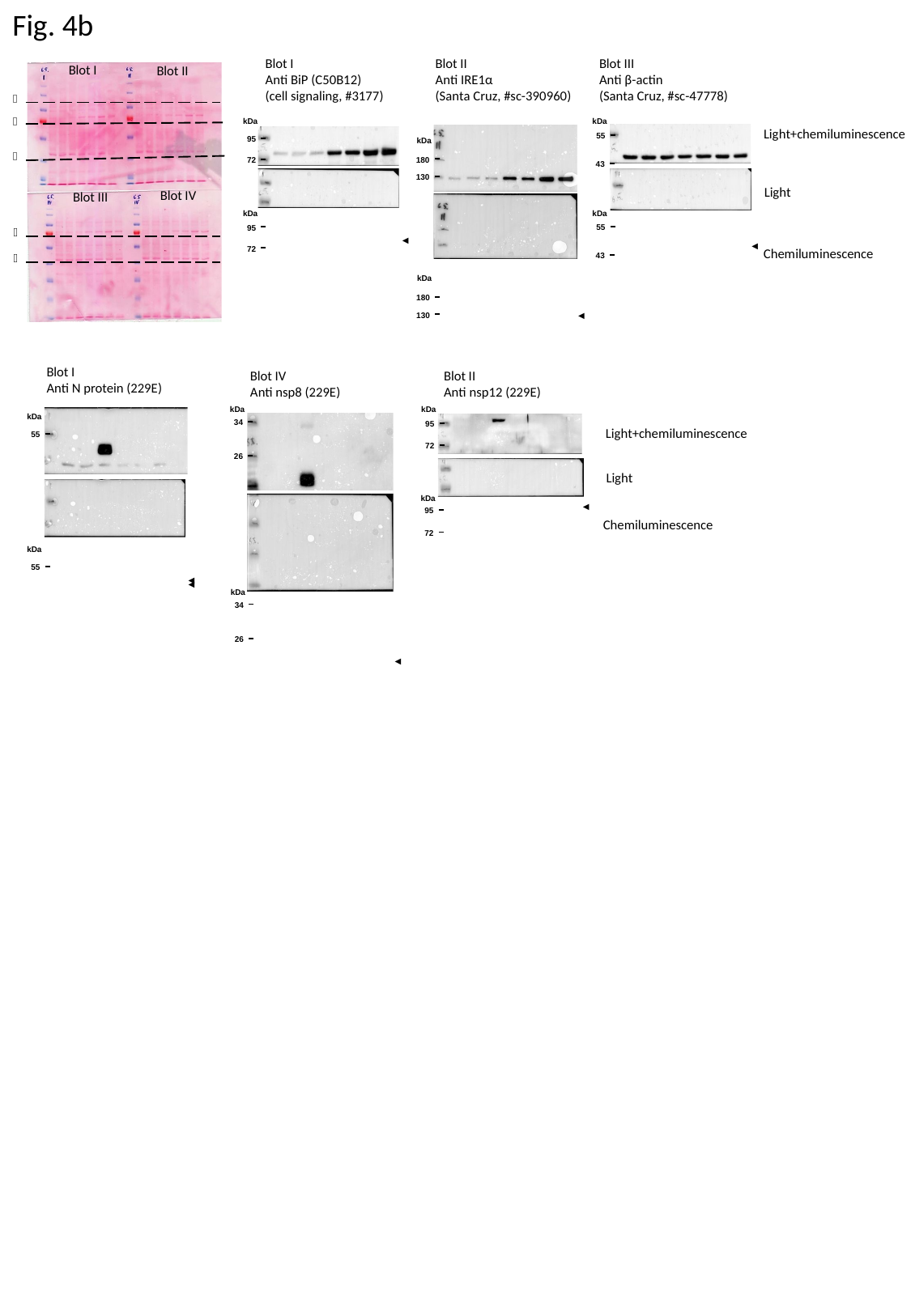

Fig. 4b
Blot III
Anti β-actin
(Santa Cruz, #sc-47778)
Blot I
Anti BiP (C50B12)
(cell signaling, #3177)
Blot II
Anti IRE1α
(Santa Cruz, #sc-390960)
Blot I
Blot II
Light+chemiluminescence
Light
Blot IV
Blot III
Chemiluminescence
Blot I
Anti N protein (229E)
Blot IV
Anti nsp8 (229E)
Blot II
Anti nsp12 (229E)
Light+chemiluminescence
Light
Chemiluminescence

## Slide 4
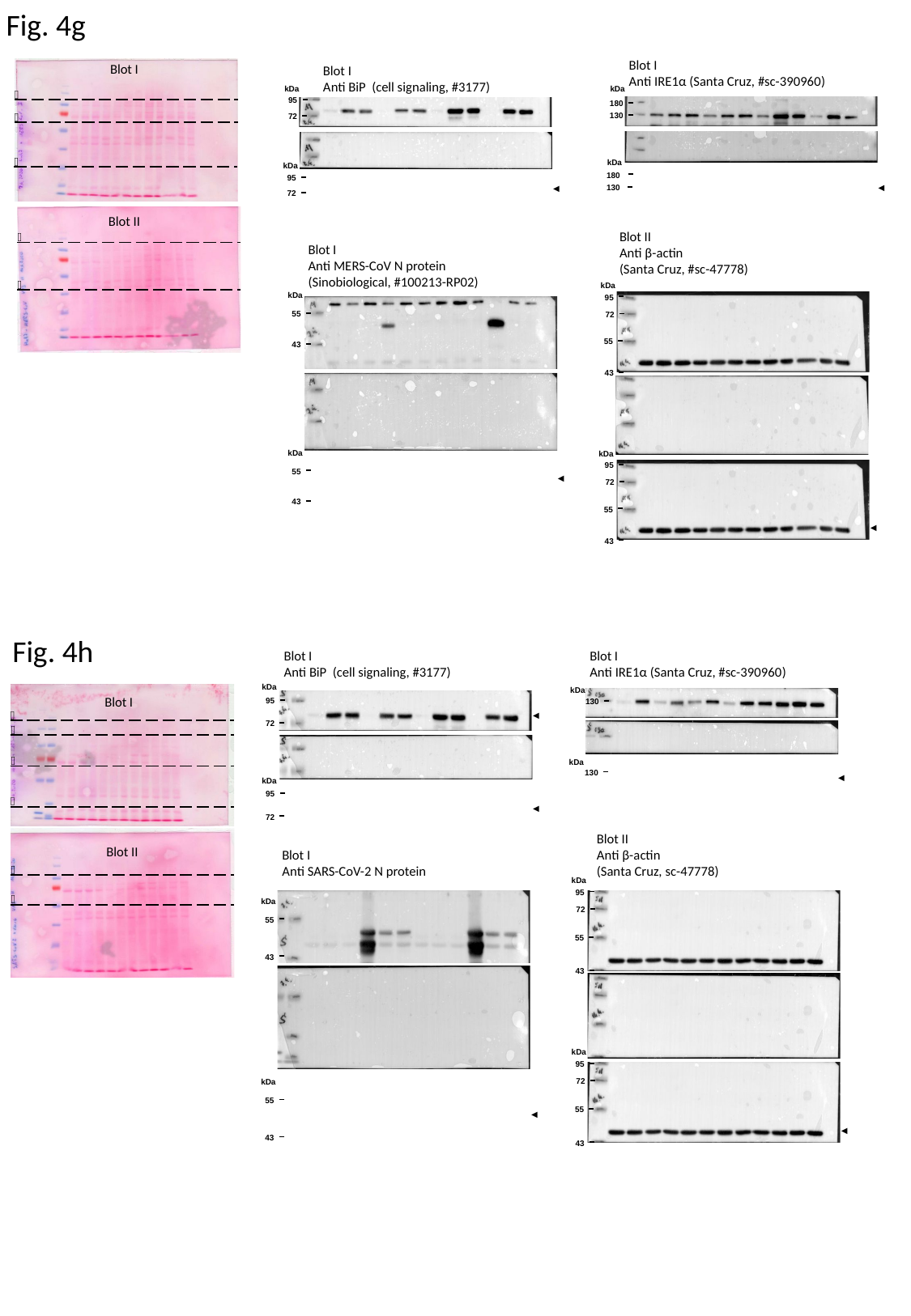

Fig. 4g
Blot I
Anti IRE1α (Santa Cruz, #sc-390960)
Blot I
Blot I
Anti BiP (cell signaling, #3177)
Blot II
Blot II
Anti β-actin
(Santa Cruz, #sc-47778)
Blot I
Anti MERS-CoV N protein
(Sinobiological, #100213-RP02)
Fig. 4h
Blot I
Anti BiP (cell signaling, #3177)
Blot I
Anti IRE1α (Santa Cruz, #sc-390960)
Blot I
Blot II
Anti β-actin
(Santa Cruz, sc-47778)
Blot II
Blot I
Anti SARS-CoV-2 N protein

## Slide 5
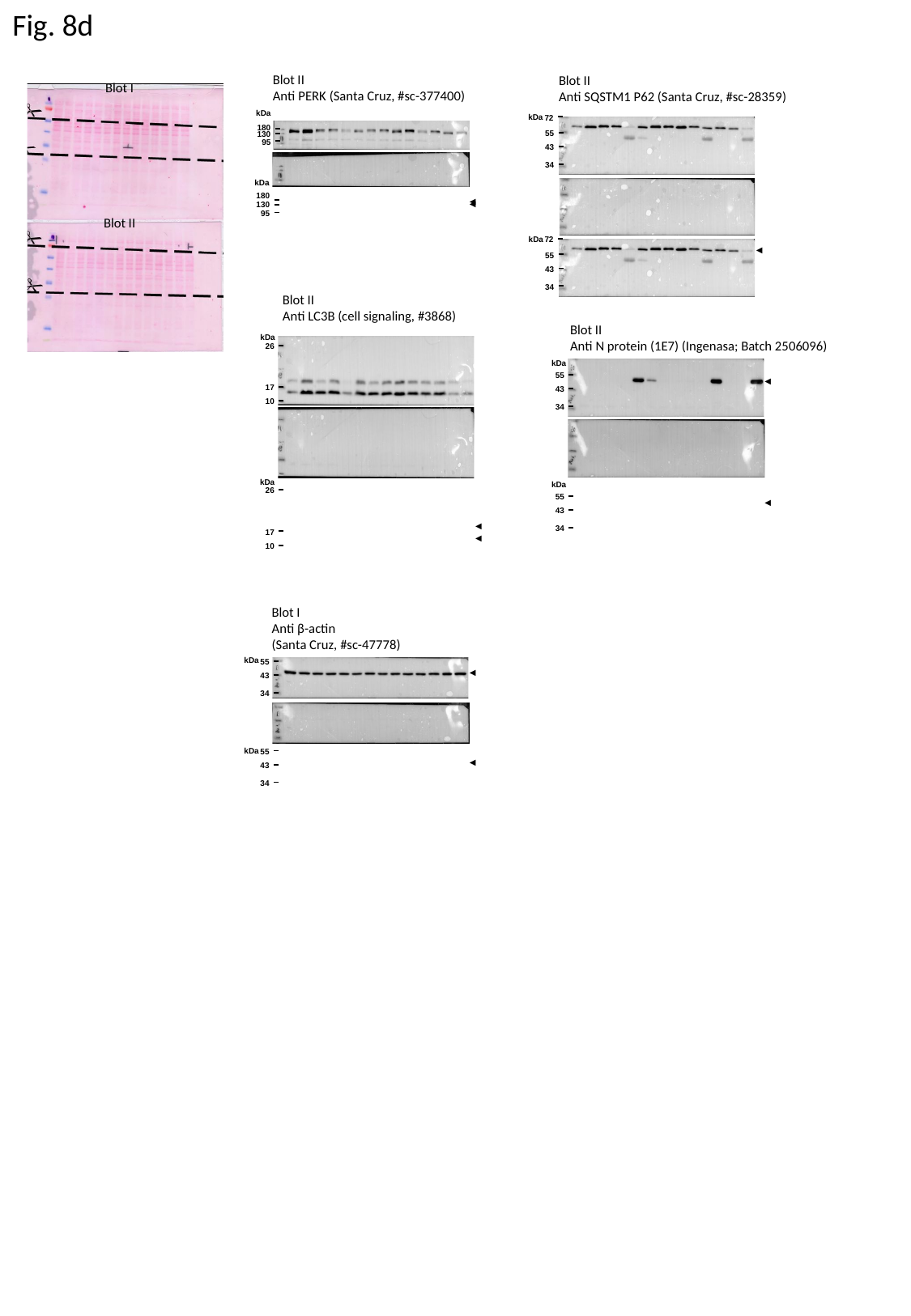

Fig. 8d
Blot II
Anti PERK (Santa Cruz, #sc-377400)
Blot II
Anti SQSTM1 P62 (Santa Cruz, #sc-28359)
Blot I
Blot II
Blot II
Anti LC3B (cell signaling, #3868)
Blot II
Anti N protein (1E7) (Ingenasa; Batch 2506096)
Blot I
Anti β-actin
(Santa Cruz, #sc-47778)

## Slide 6
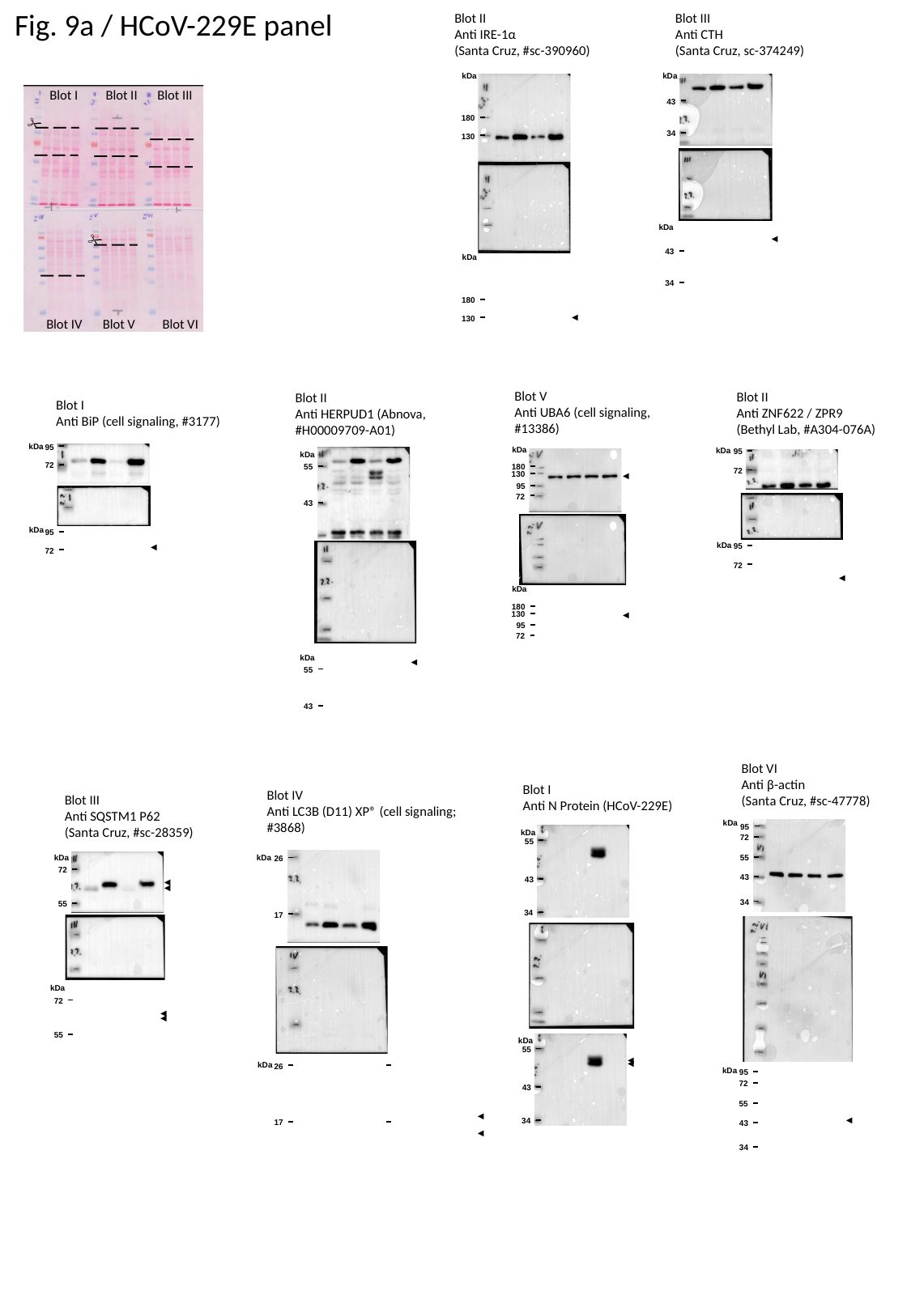

Fig. 9a / HCoV-229E panel
Blot II
Anti IRE-1α
(Santa Cruz, #sc-390960)
Blot III
Anti CTH
(Santa Cruz, sc-374249)
Blot I
Blot II
Blot III
Blot IV
Blot V
Blot VI
Blot V
Anti UBA6 (cell signaling,
#13386)
Blot II
Anti ZNF622 / ZPR9
(Bethyl Lab, #A304-076A)
Blot II
Anti HERPUD1 (Abnova,
#H00009709-A01)
Blot I
Anti BiP (cell signaling, #3177)
Blot VI
Anti β-actin
(Santa Cruz, #sc-47778)
Blot I
Anti N Protein (HCoV-229E)
Blot IV
Anti LC3B (D11) XP® (cell signaling;
#3868)
Blot III
Anti SQSTM1 P62
(Santa Cruz, #sc-28359)

## Slide 7
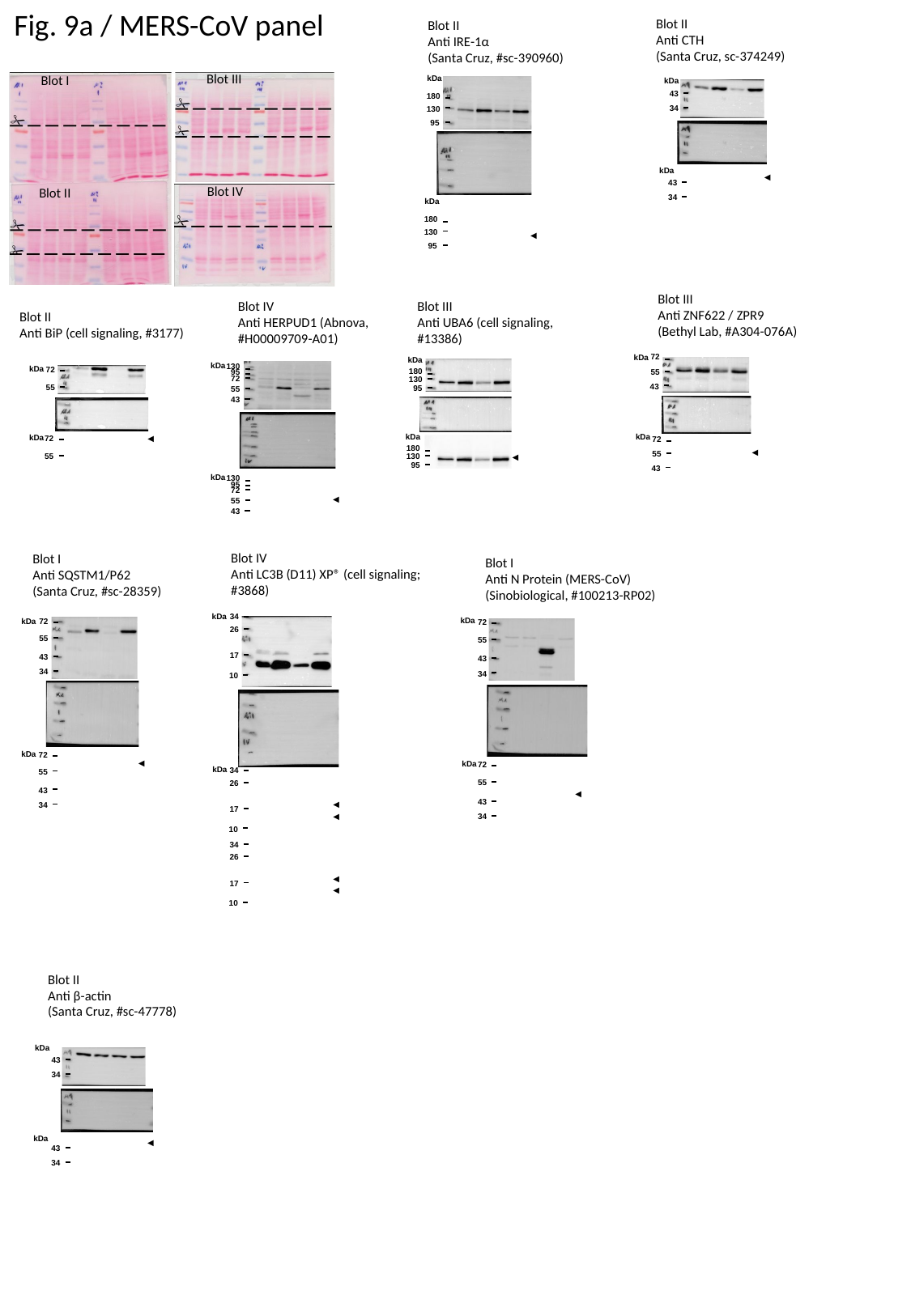

Fig. 9a / MERS-CoV panel
Blot II
Anti CTH
(Santa Cruz, sc-374249)
Blot II
Anti IRE-1α
(Santa Cruz, #sc-390960)
Blot III
Blot I
Blot IV
Blot II
Blot III
Anti ZNF622 / ZPR9
(Bethyl Lab, #A304-076A)
Blot IV
Anti HERPUD1 (Abnova,
#H00009709-A01)
Blot III
Anti UBA6 (cell signaling,
#13386)
Blot II
Anti BiP (cell signaling, #3177)
Blot IV
Anti LC3B (D11) XP® (cell signaling;
#3868)
Blot I
Anti SQSTM1/P62
(Santa Cruz, #sc-28359)
Blot I
Anti N Protein (MERS-CoV)
(Sinobiological, #100213-RP02)
Blot II
Anti β-actin
(Santa Cruz, #sc-47778)

## Slide 8
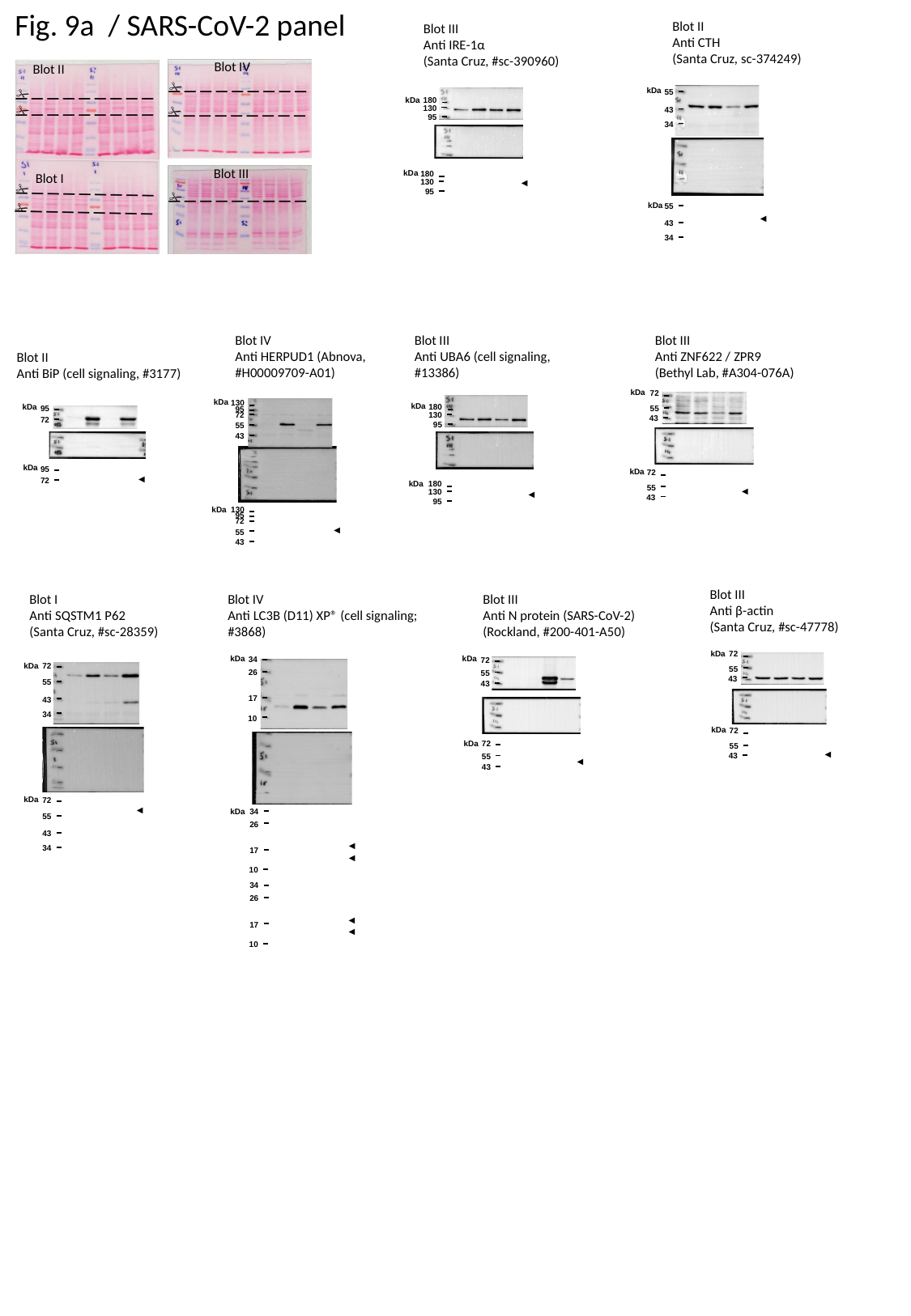

Fig. 9a / SARS-CoV-2 panel
Blot II
Anti CTH
(Santa Cruz, sc-374249)
Blot III
Anti IRE-1α
(Santa Cruz, #sc-390960)
Blot IV
Blot II
Blot III
Blot I
Blot IV
Anti HERPUD1 (Abnova,
#H00009709-A01)
Blot III
Anti UBA6 (cell signaling,
#13386)
Blot III
Anti ZNF622 / ZPR9
(Bethyl Lab, #A304-076A)
Blot II
Anti BiP (cell signaling, #3177)
Blot III
Anti β-actin
(Santa Cruz, #sc-47778)
Blot I
Anti SQSTM1 P62
(Santa Cruz, #sc-28359)
Blot IV
Anti LC3B (D11) XP® (cell signaling;
#3868)
Blot III
Anti N protein (SARS-CoV-2)
(Rockland, #200-401-A50)

## Slide 9
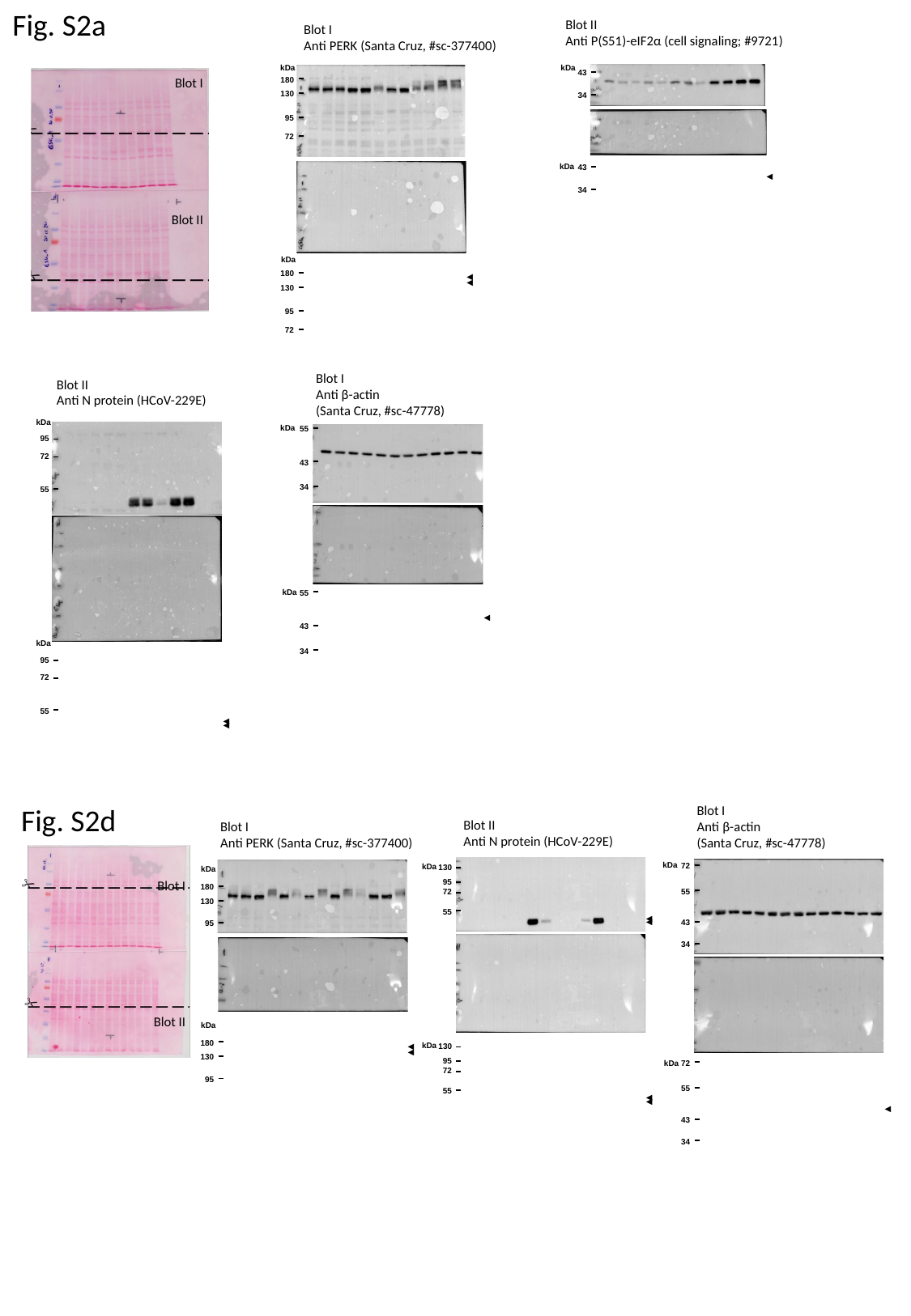

Fig. S2a
Blot II
Anti P(S51)-eIF2α (cell signaling; #9721)
Blot I
Anti PERK (Santa Cruz, #sc-377400)
Blot I
Blot II
Blot I
Anti β-actin
(Santa Cruz, #sc-47778)
Blot II
Anti N protein (HCoV-229E)
Fig. S2d
Blot I
Anti β-actin
(Santa Cruz, #sc-47778)
Blot II
Anti N protein (HCoV-229E)
Blot I
Anti PERK (Santa Cruz, #sc-377400)
Blot I
Blot II

## Slide 10
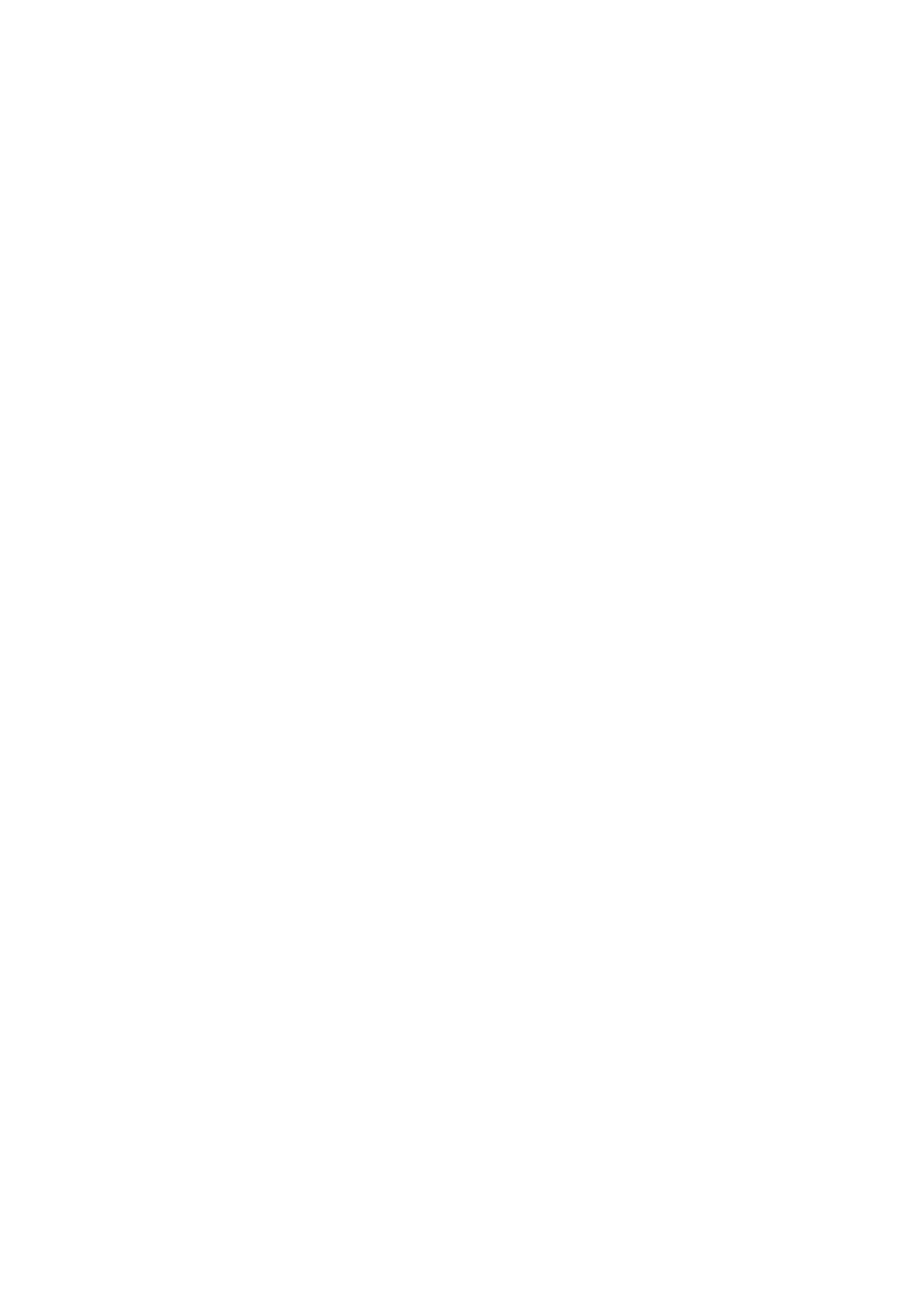

Supplement: Supplementary file 4 — Source Data [file 41467_2021_25551_MOESM4_ESM.zip › NCOMMS-20-36100A-Immunoblot_source_data_final.pptx]
